# Alpha Phase Synchronization of Parietal Areas Reflects Switch-Specific Activity During Mental Rotation: An EEG Study

**DOI:** 10.3389/fnhum.2018.00259

**Published:** 2018-06-21

**Authors:** Hiroshi Yokoyama, Isao Nambu, Jun Izawa, Yasuhiro Wada

**Affiliations:** ^1^Graduate School of Engineering, Nagaoka University of Technology, Nagaoka, Japan; ^2^Faculty of Engineering, Information and System, University of Tsukuba, Tsukuba, Japan

**Keywords:** electroencephalography, phase synchronization, weighted phase-lag index, dynamic time warping, functional connectivity, parietal cortex, action switching, mental rotation

## Abstract

Action selection is typically influenced by the history of previously selected actions (the immediate motor history), which is apparent when a selected action is switched from a previously selected one to a new one. This history dependency of the action selection is even observable during a mental hand rotation task. Thus, we hypothesized that the history-dependent interaction of actions might share the same neural mechanisms among different types of action switching tasks. An alternative hypothesis is that the history dependency of the mental hand rotation task might involve a distinctive neural mechanism from the general action selection tasks so that the reported observation with the mental hand rotation task in the previously published literature might lack generality. To refute this possibility, we compared neural activity during action switching in the mental hand rotation with the general action switching task which is triggered by a simple visual stimulus. In the experiment, to focus on temporal changes in whole brain oscillatory activity, we recorded electroencephalographic (EEG) signals while 25 healthy subjects performed the two tasks. For analysis, we examined functional connectivity reflected in EEG phase synchronization and analyzed temporal changes in brain activity when subjects switched from a previously selected action to a new action. Using a clustering-based method to identify functional connectivity reflected in time-varying phase synchronization, we identified alpha-power inter-parietal synchronization that appears only during switching of the selected action, regardless of the hand laterality in the presented image. Moreover, the current study revealed that for both tasks the extent of this alpha-power inter-parietal synchronization was altered by the history of the selected actions. These findings suggest that alpha-power inter-parietal synchronization is engaged as a form of switching-specific functional connectivity, and that switching-related activity is independent of the task paradigm.

## 1. Introduction

Action selection is realized within the conflict between a previously selected action and an alternative one. A recent study (Kent et al., 2009) suggested that a given action selection is biased toward previously selected actions, even in the absence of an explicitly sequential structure that must be learned or implemented. The effects of this bias are clearly observed when a subject must switch from a previously selected action to a different one. Thus, the history of previously selected actions (i.e., the immediate motor history) comes into play when deciding whether to repeat a previously selected action or switch to a different action, independent of the sequential nature of the task paradigm. However, since studies involving actual motor performance cannot easily dissociate the action selection process from the sensory feedback generated by motor execution, it remains unclear whether the decision regarding action selection is directly caused by motor history or by sensory feedback resulting from the motor executions.

One way of addressing this issue is to examine the neural correlates of action switching using a task that does not generate sensory feedback. One possible approach is to adopt a motor imagery task. Motor imagery is a well-known paradigm, that activates the same neural representations as motor planning, without requiring physical execution (Jeannerod, 2001; Munzert et al., 2009). We therefore predicted that a motor imagery task could dissociate the neural process of action selection from the effects of sensory feedback. A recent study by Helmich et al. (2009) examined the relationship between action selection and motor history using a motor imagery task. Specifically, they used a mental hand rotation task in which subjects were asked to judge the laterality (“handedness”) of visually rotated images of a left or right hand. This task is widely used, because experimental evidence indicates that both the reaction time for identifying the type of hand and the neural activation in motor-related brain regions are influenced by body posture and by the angle of rotation of the hand image (Cooper and Shepard, 1975; de Lange et al., 2006). Using this task, Helmich and colleagues found evidence for an interaction between action selection and motor history (Helmich et al., 2009). Given these findings regarding motor imagery, the mental hand rotation task would seem to be a useful experimental paradigm for examining the effects of motor history on action selection, in the absence of any sensory feedback associated with the motor execution.

However, although many studies have provided evidence that the mental rotation task involves the mental simulation of hand movement (Cooper and Shepard, 1975; Zacks, 2008; Osuagwu and Vuckovic, 2014), a recent cognitive study (Cona et al., 2017) offered a contrary opinion. This study reported that transcranial magnetic stimulation of the supplementary motor area in the brain affects mental rotation performance both in versions using pictures of objects as well as in versions using pictures of hands. This indicates that the cognitive process of visuospatial transformation is predominantly facilitated during the mental hand rotation task, rather than motor imagery, raising the possibility that some of the neural correlates of action switching in the mental hand rotation task (Helmich et al., 2009) are specialized for the task, and are not involved in general action switching (Kent et al., 2009).

The purpose of this study was to explore whether the history-dependence observed in the mental rotation task derives from the neural processing of general action switching. To this end, we used the mental hand rotation task and also a general action switching task referred to as the “command-to-response task,” in which subjects were instructed to judge the laterality of left or right angle brackets presented visually, without mental hand rotation. We hypothesized that the history-dependent interaction during the mental hand rotation task shares neural mechanisms with other types of action switching triggered by simple visual stimuli (such as the command-to-response task). If there are common neural mechanisms of action switching underlying the performance of these two tasks, similar neural activity would be expected in both tasks. To test this hypothesis, we compared the neural activity generated by both tasks.

**Figure 1:**
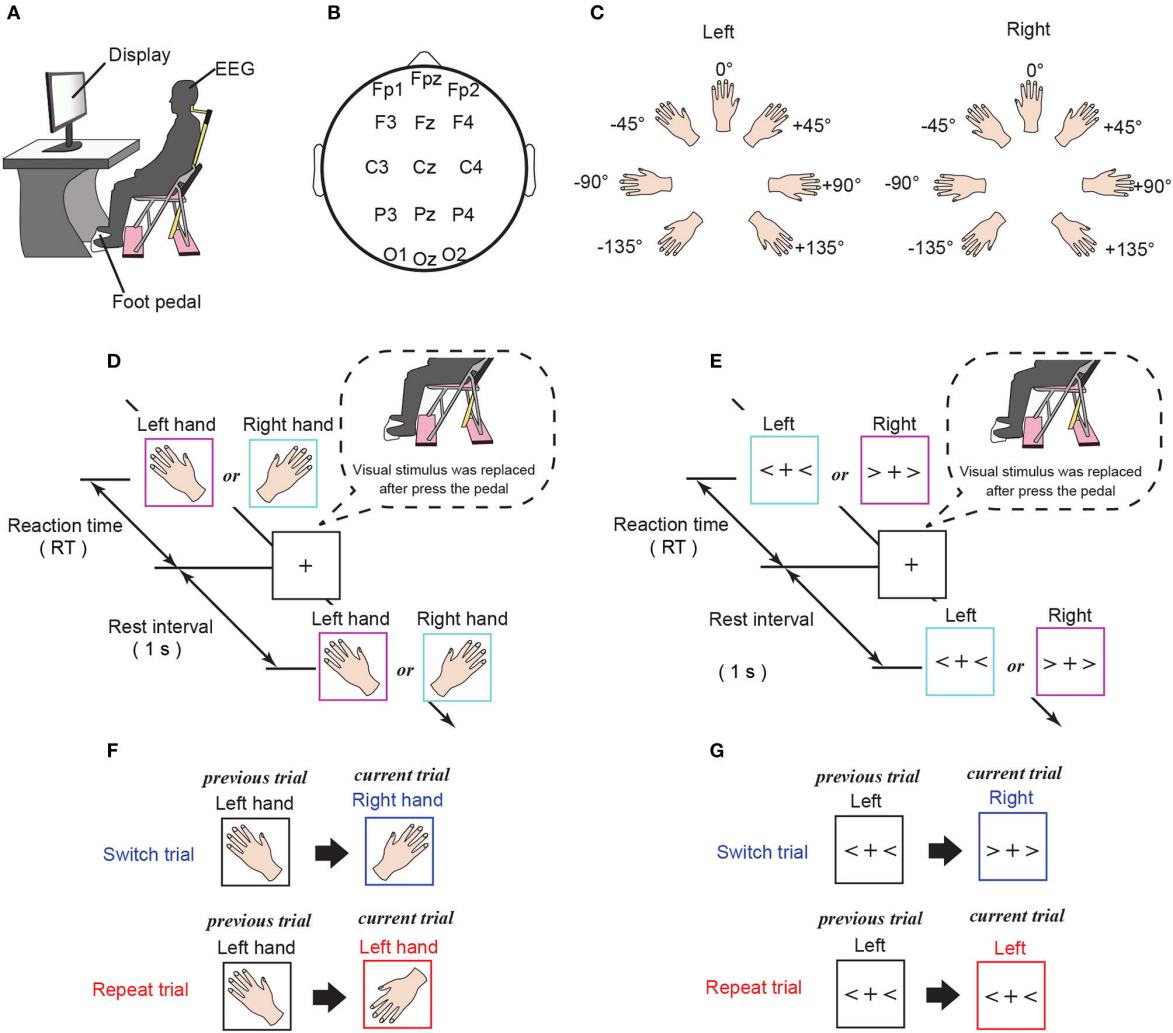
Experimental settings. **(A)** Experimental environment. Subjects were instructed to judge the hand laterality of an image presented on a monitor. **(B)** Channel location. Fifteen standard scalp electrodes, specifically, Fp1, Fpz, Fp1, F3, Fz, F4, C3, Cz, C4, P3, Pz, P4, O1, Oz, and O2, were selected using the International 10–20 system. **(C)** Rotating pattern of presented stimuli for the mental hand rotation task. **(D)** Experimental procedures for the mental hand rotation task. Images of a left or right hand were randomly presented on the computer monitor and shown until the subject made a response. To report the response, subjects were required to press the pedal with the foot corresponding to the laterality of the presented stimuli. After the response, the stimulus was replaced by a fixation cross, which was shown for 1 s until the next stimulus was presented. **(E)** Experimental procedures for the command-to-response task. Left or right angle brackets were randomly presented on the computer monitor and shown until the subject made a response. To report the response, subjects were required to press the pedal with the foot corresponding to the laterality of the presented stimuli. After the response, the stimulus was replaced by a fixation cross, which was shown for 1 s until the next stimulus was presented. **(F,G)** Definition of the trial-type (switch or repeat) for each task.

In the experiment, in order to focus on temporal changes in brain activity during the tasks, we measured electroencephalographic (EEG) signals while 25 healthy subjects performed each task (Figures 1A,D–G). We examined the temporal effects of motor history in functional brain structures exhibiting time-varying phase oscillatory activity, using a data-driven method to compare brain activity in trials containing identical (repeat trial) and different (switch trial) stimuli with respect to the previous trial.

## 2. Materials and methods

### 2.1. Procedures and designs

#### 2.1.1. Mental hand rotation task

Twenty-five healthy right-handed males (age range: 21–29 years, mean 23.5) participated in the experiments. The Ethics Committee of the Nagaoka University of Technology approved this study. All procedures were performed in accordance with the Declaration of Helsinki. All subjects provided written informed consent before participating in the experiments.

In each experimental session, the subject was asked to sit in a chair placed in front of a computer monitor (Figure 1A). After viewing a visual stimulus (a picture of a left or right hand), subjects were instructed to press a foot pedal with the foot corresponding to the same side of the body as the visual stimulus. Responses were given by foot rather than hand in order to minimize, as much as possible, any potential overlap of the neural processes of the mental simulation triggered by the mental rotation and the motor execution required for reporting the response. Each visual stimulus was displayed until the subject pressed the foot pedal. Following a 1 s rest interval, during which subjects were instructed to gaze at a fixed point, the next visual stimulus was presented (Figure 1D). In the mental hand rotation task, each stimulus was randomly selected from 14 hand images (2 hands × 7 angles) and subjects were instructed to judge the laterality of the hand (see Figures 1C,D). We used two trial types (Figure 1F): (i) trials with stimuli that were identical to the previous trial (repeat trials), and (ii) trials with stimuli that were different from those in the previous trial (switch trials). The visual stimuli were presented once per trial and were controlled so that the frequency of repeat trials was approximately 60%. Each experimental session included 112 trials, and each subject completed 10 experimental sessions.

We measured 64-channel scalp EEG for each subject (Active Two; Biosemi, Amsterdam, the Netherlands; sampling frequency: 1,024 Hz) during the task. For analysis, the 15 channels typically corresponding to the frontal cortex (Fp1, Fpz, Fp1, F3, Fz, and F4), motor cortex (C3, Cz, and C4), parietal cortex (P3, Pz, and P4) and visual cortex (O1, Oz, and O2) were manually selected according to the International 10–20 system (Figure 1B, Koessler et al., 2009). The channels over the frontal and parietal cortices (Fp1, Fpz, Fp1, F3, Fz, F4, P3, Pz, and P4) were selected because the parieto-frontal region plays an important role in task selection and decision-making (Cisek, 2006; Hare et al., 2011; Tosoni et al., 2014). We selected channels over the motor and visual cortices (C3, Cz, C4, O1, Oz, and O2), because these regions are involved in the neural processes underlying mental hand rotation (Chen et al., 2013; Horst et al., 2013) and visual mental rotation (Podzebenko et al., 2002). In addition, for each trial, we measured the time interval between the appearance of the visual stimulus and the foot response from the subject, which was defined as the reaction time (RT). We then used the RT data to evaluate behavioral performance.

#### 2.1.2. Command-to-response task

The action selection task without mental hand rotation was conducted with the same subjects and experimental environment as the mental hand rotation task, except that the experiment took place on a different day. Left or right angle brackets were presented as visual stimuli (Figures 1E,G). In each trial, the visual stimuli were shown until a foot pedal was pressed, followed by a rest interval of 1 s before the presentation of the next visual stimulus (Figure 1E). Subjects were asked to judge the laterality of each angle bracket (left or right), using a foot pedal, as described above. The visual stimuli were presented once per trial, and the stimuli were controlled so that the frequency of the repeat trials was approximately 60%. Visual stimuli were presented in 112 trials per session, and each subject completed six sessions. We measured RTs and collected 64-channel scalp EEG for each subject during the experiment.

### 2.2. Behavioral analysis

Behavioral analysis was conducted using Matlab (Mathworks, Natick, MA, USA). To identify and reject trials with outlier RTs, the distribution of RTs for each subject across all sessions was fitted using ex-Gaussian functions (Baayen and Milin, 2010; Matzke et al., 2013). We excluded data exceeding the 95th percentile confidence interval, as outlier trials. Moreover, trials containing an incorrect response were also excluded from the analysis. Using this procedure, for each subject, we rejected an average of 11.66 ± 5.51% (mean ± standard deviation, across subjects) of the trials in the mental hand rotation task, per subject. As a result, for each orientation angle of this task, an average of 86.45 ± 5.34% of the switch trials, and 89.58 ± 4.73% of the repeat trials, were analyzed for each subject. Similarly, in the command-to-response task, we rejected 6.80 ± 1.83% of the trials, and as a result, 93.15 ± 2.57% of the switch trials and 93.22 ± 2.08% of the repeat trials were analyzed for each subject. After rejecting outliers, we also analyzed the mean RTs.

For the mental hand rotation task, we examined three factors: hand laterality (left or right), angle of presentation, and trial type (switch or repeat). For quantitative analysis, we used a three-way analysis of variance (ANOVA) with motor history (repeat or switch), angle (−135°, −90°, −45°, 0°, +45°, +90°, or +135°), and hand laterality (left or right) as factors. In addition, to determine the difference in the behavioral performance based on the task paradigm, we compared the behavioral data in the command-to-response task (switch, repeat) with the mental hand rotation task (switch, repeat), using a paired *t*-test.

### 2.3. EEG analysis

We conducted EEG analyses for both tasks using Matlab. The procedures in the following subsections were applied for the analysis of three frequency bands (alpha: 8–15 Hz, beta 1: 16–24 Hz, and beta 2: 28–36 Hz) for all tasks and all conditions (trial type: switch/repeat; hand- laterality: left/right). We focused primarily on the alpha band in this study, as a recent study showed that the alpha oscillation mediates perceptual switching (Matsuda et al., 2017). The results for the other frequency bands can be found in the Supplementary Materials.

#### 2.3.1. Preprocessing

The duration of single epochs of the EEG data included the interval from −0.4 s to 1.2 s (with the visual stimulus occurring at 0.0 s). The EEG data in each epoch were filtered between 1–100 Hz with a 3rd order Butterworth filter, with baseline correction. Baseline was defined as the immediate pre-stimulus period (equal to 0.4 s before stimulus presentation) in each trial. We conducted this preprocessing procedure for both the mental hand rotation task and the command-to-response task, prior to evaluating phase synchronization and functional connectivity. Note that the EEG data corresponding to the excluded trials in the behavioral analysis were also removed from the EEG analysis. To conduct the preprocessing, we applied the “butter()” and “filtfilt()” functions in the “Signal Processing Toolbox” in MATLAB.

#### 2.3.2. Phase synchronization

We examined temporal changes in inter-regional phase synchronization reflected in the scalp EEG recordings, based on two findings from recent studies: (i) phase synchronization in EEG was reported to be related to the exact timing of neural communication among different brain areas (Varela et al., 2001; Sauseng and Klimesch, 2008), and (ii) alpha oscillation was found to play a role in perceptual switching during a cognitive task (Matsuda et al., 2017). Furthermore, as an evaluation index for temporal changes in inter-regional phase synchronization, we used the weighted phase-lag index (wPLI), which enables analysis of the properties of phase synchronization without the deleterious impact of volume conduction (Vinck et al., 2011; Cohen, 2015). Here, we assumed that wPLIs could be used to quantify the neural dynamics reflected in the phase oscillatory activity of the scalp EEGs, independent of the effects of artifact and volume conduction. Based on the following equation for calculating wPLI, we evaluated the time varying phase oscillatory activity reflected in the scalp EEGs for each laterality condition (left and right) in accordance with the trial conditions (switch and repeat trials).

The wPLIs were computed for each EEG channels across the other 14 channels based on the following equation:

(1) $$\begin{array}{r} wPLI_{i,j}=\frac{\left|E\left\{ℑ\left\{X_{i}X_{j}^{*}\right\}\right\}\right|}{E\left\{\left|ℑ\left\{X_{i}X_{j}^{*}\right\}\right|\right\}}=\frac{\left|E\left\{|ℑ\left\{X_{i}X_{j}^{*}\right\}|\text{sgn}\left[ℑ\left\{X_{i}X_{j}^{*}\right\}\right]\right\}\right|}{E\left\{\left|ℑ\left\{X_{i}X_{j}^{*}\right\}\right|\right\}} \end{array}$$

where *i, j* are channel indices, *X*_*i*_ is the time-frequency spectrum of channel *i*, $X_{j}^{*}$ is the complex conjugate of *X*_*j*_, $ℑ\left\{X_{i}X_{j}^{*}\right\}$ indicates an imaginary section of cross frequency spectra $X_{i}X_{j}^{*}$, *E*{·} is the expected value operator, and sgn{·} is the sign function operator.

After completing the preprocessing procedure above, we used a Morlet wavelet approach to calculate the time-frequency spectrum over all EEG channels for each subject using the EEGLAB toolbox (https://sccn.ucsd.edu/eeglab/). To capture the temporal features of phase synchronization during evaluation of wPLIs, we employed a time-frequency spectrum with three different frequency bands (alpha: 8–15 Hz, beta 1: 16–24 Hz, and beta 2: 28–36 Hz) over all channel pairs for each subject. Consequently, we obtained time-varying wPLIs in each EEG channel across the other 14 channels (i.e., the total number of channel pairs was 105, 15 × [15−1]/2 = 105 pairs) for each subject. All wPLIs were then standardized for each subject using z-scores, in the following manner. First, the mean value μ_*B*_*i,j*__ in the pre-stimulus period (the 0.4 s period before the presentation of stimuli) was subtracted from the wPLIs for each channel pair. These values were then divided by the standard deviation σ_*B*_*i,j*__ of wPLI in the pre-stimulus period (Vinck et al., 2011):

(2) $$\begin{array}{r} z-wPLI_{i,j}=\frac{wPLI_{i,j}-\mu_{B_{i,j}}}{\sigma_{B_{i,j}}} \end{array}$$

The resulting z-wPLIs reflect task-related changes in synchronization compared with the pre-stimulus interval. Positive values are indicative of increased synchronization.

#### 2.3.3. Functional connectivity and clustering

**Figure 2:**
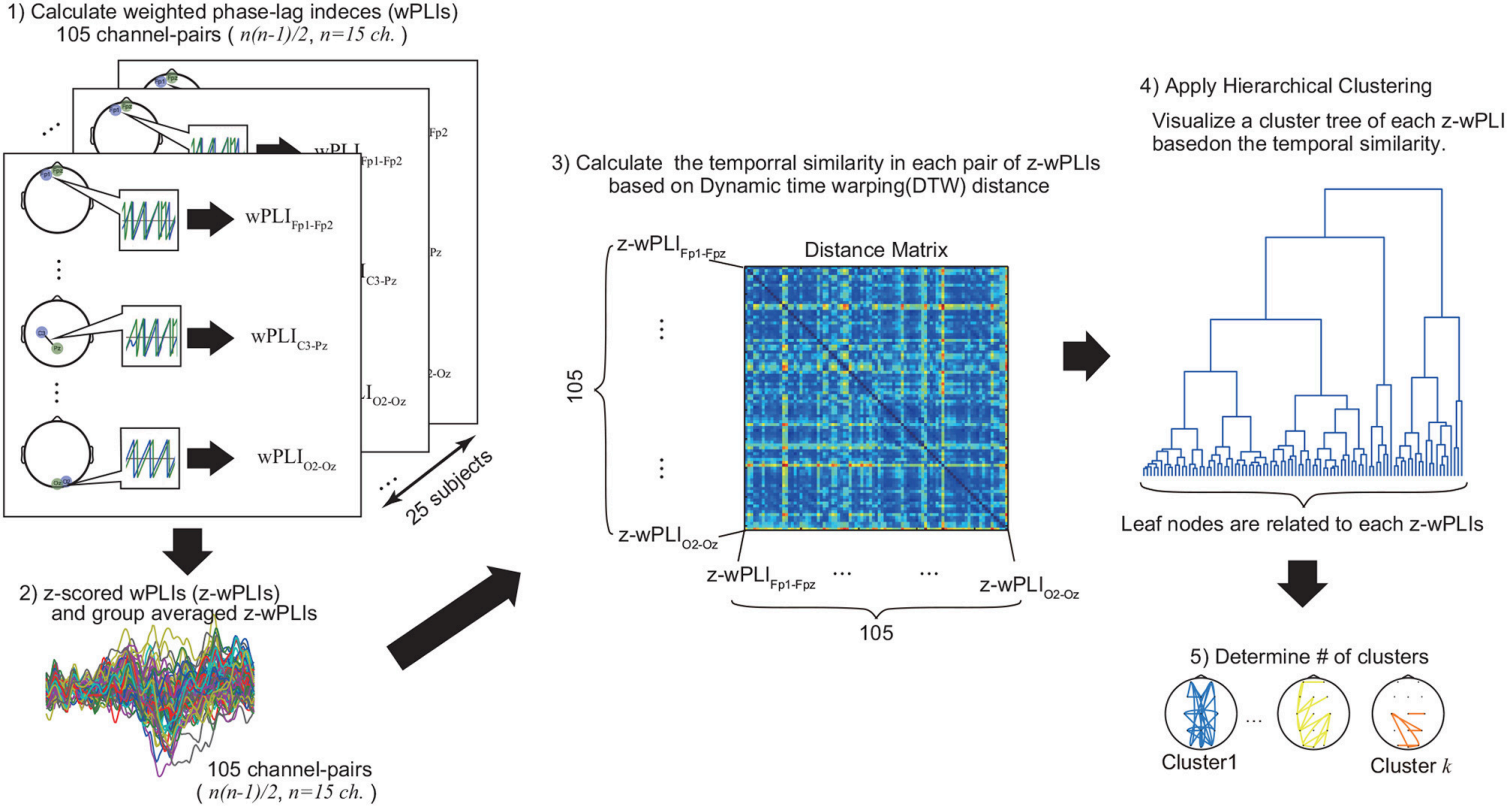
Estimation of functional connectivity. First, we computed the z-scored wPLI for each channel pair (105 pairs), then calculated the group-averaged values. Second, we evaluated the distance matrix using the DTW algorithm (see Materials and Methods section for detail). Finally, applying hierarchical clustering, we visualized the functional structures and cluster-averaged time-course of z-scored wPLIs for each identified cluster. These procedures were applied to each trial condition (switch or right) depending on the laterality condition (left or right).

Because several neurophysiological studies have reported that phase synchronization in EEG is relevant to the exact timing of neural communication among different brain areas (Varela et al., 2001; Sauseng and Klimesch, 2008), we expected that the task-related functional distribution distinct from background activity could be estimated by considering the cluster of brain structures exhibiting temporal similarities in phase synchronization. Here, we propose a method for clustering-based functional connectivity analysis, which we used to identify the functional brain structures contained in the time-varying phase oscillatory activity in a data-driven manner, as shown in Figure 2. This proposed method was employed for each trial condition (switch or repeat trial) depending on the laterality condition (left or right). Using this method, we focused primarily on the functional structure of the alpha oscillatory activity, because it has been found to play an important role in perceptual switching during cognitive tasks (Matsuda et al., 2017). The details of this proposed method are described below.

First, to create a distance matrix as an input value for hierarchical clustering, we applied the dynamic time warping (DTW) algorithm (Müller, 2007; Karamzadeh et al., 2013; Meszlényi et al., 2016) (see Supplementary Materials for details) to dynamically capture the time course similarity of the z-wPLI values. The DTW is a well-known algorithm used to compare temporal similarity between two different signals (Müller, 2007; Karamzadeh et al., 2013; Meszlényi et al., 2016). Thus, by applying the DTW algorithm to evaluate the time-course similarity in each pair of z-wPLIs, our method enabled an evaluation of the clusters containing the time-varying phase oscillatory activity based on the optimal distance. For a demonstration of the validity of the proposed method using simulated data with a phase oscillation model, see the Supplementary Materials online (see Figures S2–S4).

The clustering procedure for our proposed method is described below:

1. Compute the group-averaged values of z-wPLI for each pair of EEG channels (105 pairs).
2. Evaluate the time-course similarity in each z-wPLI using the DTW algorithm to create a distance matrix as an input value for hierarchical clustering (105 × 105 matrix).
3. Apply the hierarchical clustering (Shimaoka et al., 2010) with the average linkage algorithm.
4. Estimate the number of clusters in the cluster tree, which is determined in step 3.
5. Visualize the functional structures and cluster-averaged time-course of the z-wPLIs for each identified cluster.

In step 1, since the z-scored wPLIs (z-wPLIs) were calculated for each channel pair (105 total channel pairs), each value of the group-averaged z-wPLIs indicated the temporal changes of phase synchronization between two different channels. Afterwards, in step 2, we applied the DTW algorithm to evaluate the time-course similarity for each z-wPLIs in the interval from 0.0 to 1.2 s, and, as a result, the 105 × 105 distance matrices among each value of the group-averaged z-wPLIs were associated as input features for the hierarchical clustering in step 3. In step 3, the average linkages of these input features were visualized as a cluster tree by hierarchical clustering (see Figure 2). This visualized cluster tree indicates the hierarchical structures of the group-averaged z-wPLIs, based on the time-course similarity with the DTW algorithm, and each leaf node of this cluster tree is related to each group-averaged z-wPLIs.

To estimate the number of clusters in step 4, we applied the *PseudoF* index (Caliński and Harabasz, 1974):

(3) $$\begin{array}{r} PseudoF=\frac{SS_{B}}{SS_{W}}\times \frac{k-1}{n-k} \end{array}$$

where *SS*_*B*_ is the overall between-cluster variance of input features for hierarchical clustering, *SS*_*W*_ is the overall within-cluster variance of input features for hierarchical clustering, *k* is the number of clusters, and *n* is the number of time-course data points for z-scored wPLIs.

The *PseudoF* (Caliński and Harabasz, 1974) describes the ratio of between-cluster variance to within-cluster variance. Higher values on this index indicate greater cluster separation. The number of clusters was estimated from 1 to 10, with 10 representing the highest value on this index.

As a result, considering the corresponding channel pairs in each z-wPLI, including the clusters determined with the above procedure, we visualized the topography of functional connectivity for each cluster, and evaluated the cluster-averaged z-wPLI for each cluster (step 5). Further, we calculated the stimulus-locked and response-locked average values of z-wPLI in each cluster. To additionally consider the effect of task-onset and motor execution, we considered temporal changes of z-wPLI for both stimulus-locked and response-locked average values.

These procedures were employed for EEG data analysis for each trial condition (switch and repeat trial) depending on the laterality condition (left and right) in both tasks (mental hand rotation and command-to-response task).

#### 2.3.4. Similarity analysis

To evaluate the cluster-by-cluster similarity of the alpha band connectivity patterns, we applied the cosine similarity method (Mars et al., 2016), defined as follows:

(4) $$\begin{array}{r} sim\left(A,B\right)=\frac{\sum_{i,j}a_{i,j}\times b_{i,j}}{\sqrt{\sum_{i,j}{\left(a_{i,j}\right)}^{2}\times \sum_{i,j}{\left(b_{i,j}\right)}^{2}}} \end{array}$$

where *A* and *B* denote the adjacency matrices associated with the connectivity pattern for each cluster to be evaluated, and *a*_*i,j*_ and *b*_*i,j*_ are the elements of the adjacency matrix with index number *i, j*.

We then considered similarity in two conditions: (1) the similarity between the two laterality conditions (mental rotation task: left hand vs. right hand/command-to-response task: left angle bracket vs. right angle bracket) in each task for switch trials, and (2) the similarity between the two tasks with the same laterality condition (left hand vs. left angle bracket/right hand vs. right angle bracket).

#### 2.3.5. Statistical test of evaluated clusters

We applied the surrogate method (Lachaux et al., 1999) to test the statistical significance of the cluster-averaged z-wPLI data. The surrogate data for the cluster-averaged responses were constructed based on the following procedures.

First, we calculated randomized wPLIs for all channel pairs for each subject. These randomized data were created by shuffling the time samples within one of the two channels. The randomized wPLIs for all channel pairs were standardized (z-scored) for each subject (see Equation 2), using the same procedure as for the actual data. These z-scored values of randomized time series data were averaged over all subjects. Following these steps, we applied the same clustering procedure used for the actual EEG data to the randomized data, in order to calculate the cluster-averaged data. Finally, we repeated these procedures 50 times, and all of the resulting samples of random cluster-averaged z-wPLIs were used to construct the null distribution. This enabled us to test the actual cluster-averaged z-wPLI data, as the total number of samples was over 15,000.

For statistical analysis of the cluster-averaged data for the z-scored wPLI, we used the null distribution to estimate the threshold (*p* = 0.05). To examine the effects of multiple comparisons, all statistical test results were corrected using the false discovery rate (FDR) method (Benjamini and Hochberg, 1995).

## 3. Result

### 3.1. Behavioral result

We first computed the RTs to evaluate how motor history affects the neural process associated with selecting an action in healthy subjects.

**Figure 3:**
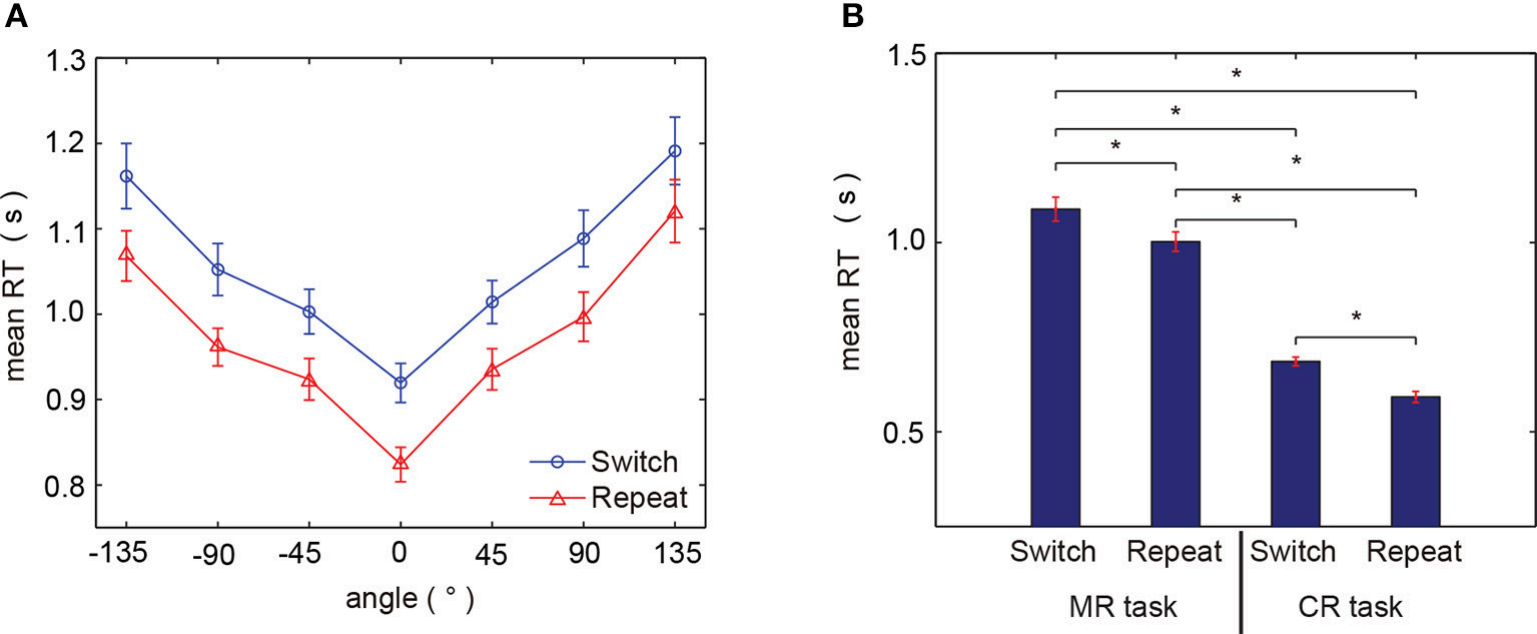
Behavioral results. **(A)** Mean RTs for all subjects for each presented angle (red: repeat trial, blue: switch trial). **(B)** Comparison of RTs between the two tasks for each trial type. MR task and CR task refer to the mental hand rotation task and command-to-response task, respectively. Asterisks (*) indicate *p* < 0.05 by paired *t*-test with FDR correction. Error bars indicate the standard error of the mean.

The mean RTs across subjects for each of the angles presented exhibited a significant orientation effect, specifically, RTs increased as the hand angle deviated from 0° (Figure 3A). This RT orientation effect is consistent with previous studies (Cooper and Shepard, 1975; Kosslyn et al., 1998; de Lange et al., 2006; Thayer and Johnson, 2006; ter Horst et al., 2010; Horst et al., 2012), where increased RTs are associated with increased angles of mental rotation imagined of their own hands, in order to match the portrayed one. Thus, this behavioral tendency observed in the current study confirmed that mental hand rotation was indeed engaged during our task. Moreover, RTs in the switch trials were greater than those in the repeat trials, and trial-related differences in RT were generally constant across all presented angles. Statistical analyses showed that RTs varied significantly according to three factors: angle, hand laterality, and trial type [trial type: *F*_(1, 691)_ = 51.73, *p* < 0.0001, η_*p*_ = 0.070; angle order: *F*_(6, 691)_ = 36.17, *p* < 0.0001 η_*p*_ = 0.239; hand order: *F*_(1, 691)_ = 10.48, *p* < 0.01 η_*p*_ = 0.015, by three-way ANOVA]. Further, as shown in Figure 3B, the mean RTs in the command-to-response task (mean ± standard error of mean across subjects; switch trials: 0.6860 ± 0.0117 s, repeat trials: 0.5922 ± 0.0147 s) were shorter than those observed in the mental hand rotation task (averaged over all subjects; switch trials:1.0880 ± 0.0316 s, repeat trials: 1.0023 ± 0.0277 s), comparing the group averaged RTs, and based on the trial conditions for each task. All paired comparisons between averaged reaction times in each task condition were found to be significantly different (*p* < 0.05 by paired *t*-tests, with FDR, Figure 3B).

### 3.2. Clustering analysis: mental hand rotation task

**Figure 4:**
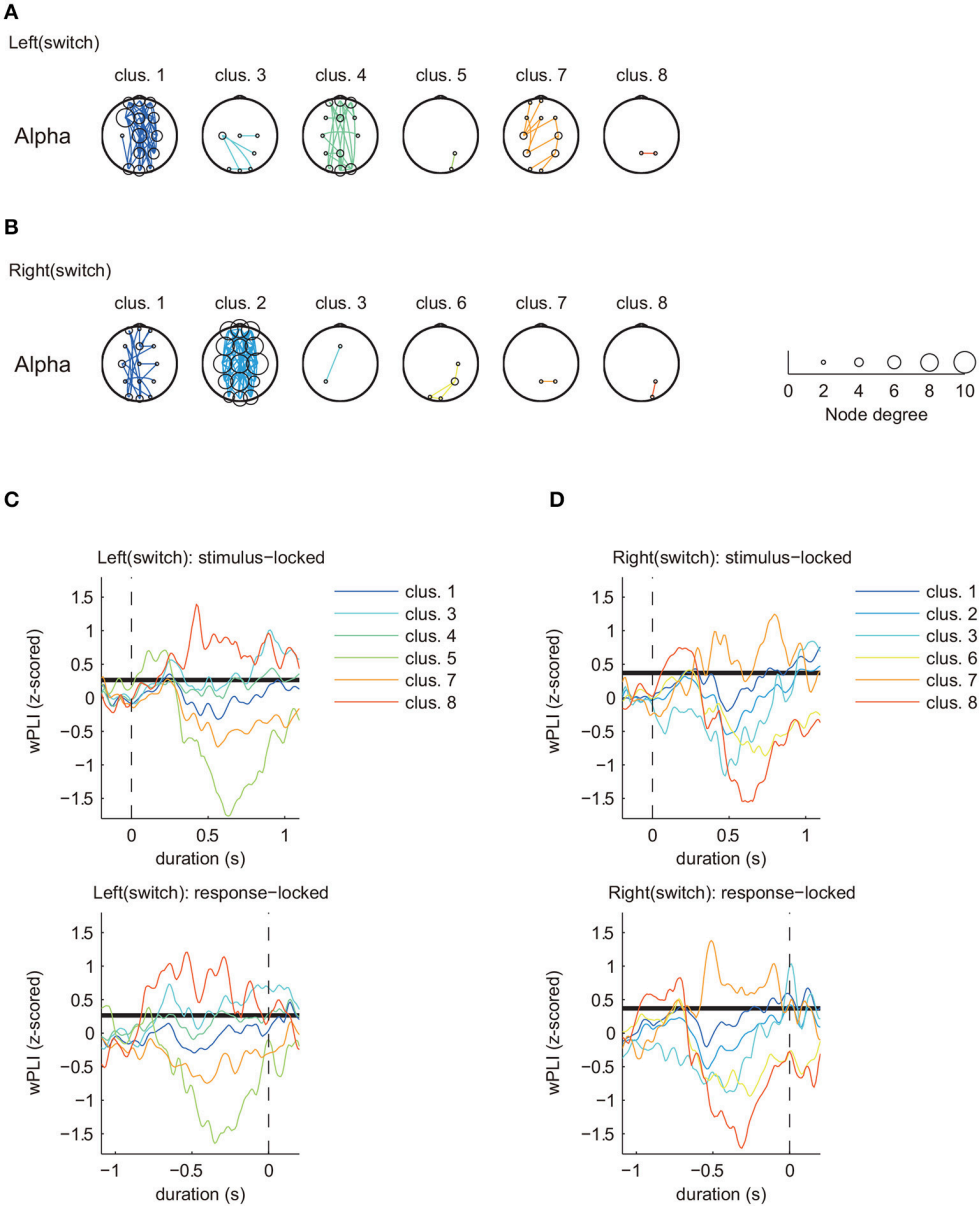
Estimated clusters and functional connectivity based on time series matching of z-scored wPLIs using DTW (MR task: switch trial, alpha band). **(A)** Estimated significant clusters of functional connectivity for left switch trials. Six of eight clusters were estimated to be significant. **(B)** Estimated significant clusters of functional connectivity for right switch trials. Six of eight clusters were estimated to be significant. Marker size of the electrodes for each topographical location corresponds with the node degree of connectivity. **(C,D)** Cluster-averaged z-scored wPLIs for each hand in the switch trials (upper panels: stimulus-locked average; lower panels: response-locked average). Bold black lines indicate a significant level of temporal changes in cluster-averaged phase-synchronization values (*p* < 0.05 with FDR correction).

**Figure 5:**
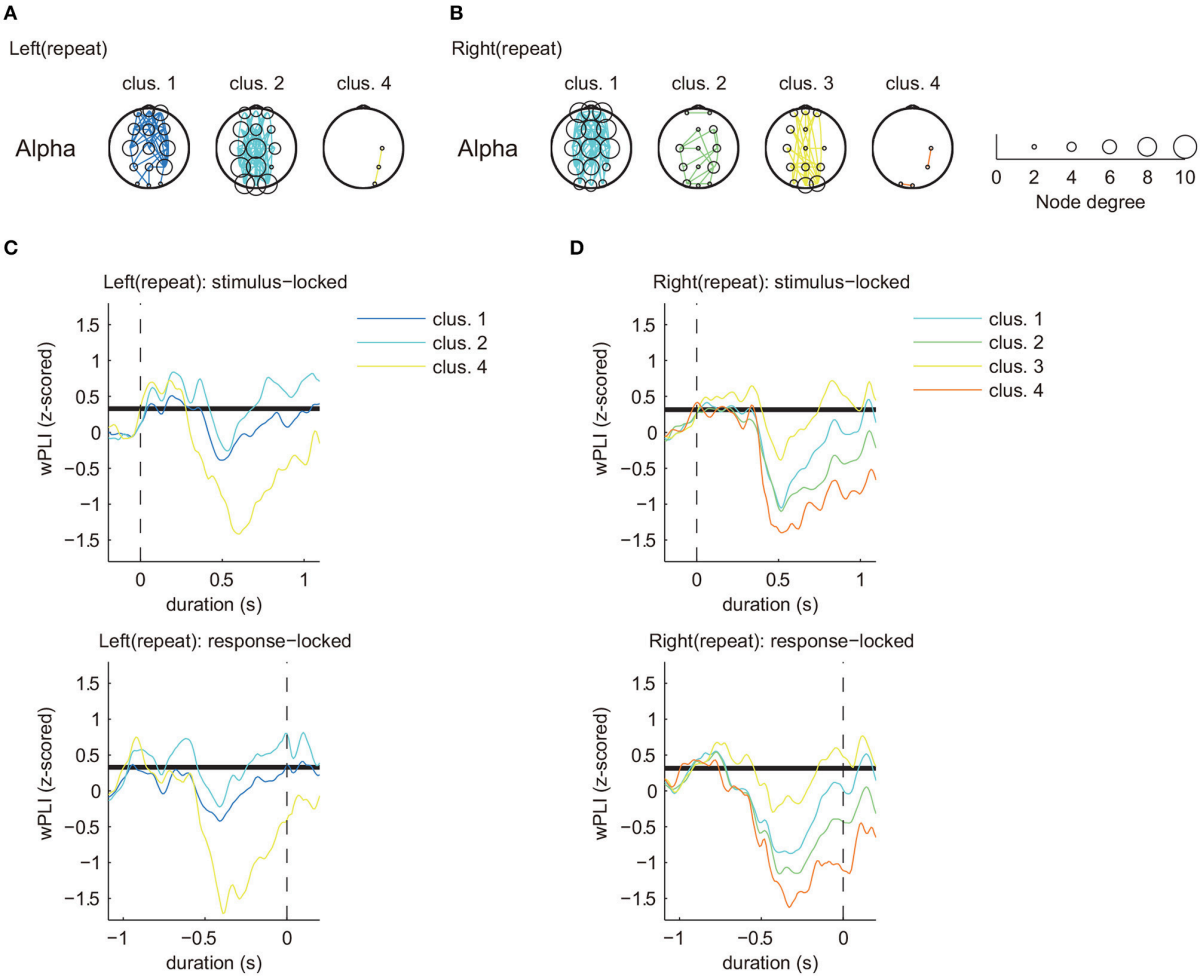
Estimated clusters and functional connectivity based on time series matching of z-scored wPLIs using DTW (MR task: repeat trial, alpha band). **(A)** Estimated significant clusters of functional connectivity for left repeat trials. Three of five clusters were estimated to be significant. **(B)** Estimated significant clusters of functional connectivity for right repeat trials. All four clusters were estimated to be significant. Marker size of the electrodes for each topographical location corresponds to the node degree of connectivity. **(C,D)** Cluster-averaged z-scored wPLIs for each hand in the repeat trials (upper panels: stimulus-locked average; lower panels: response-locked average). Bold black lines indicate a significant level of temporal changes in cluster-averaged value of phase-synchronization (*p* < 0.05 with FDR correction).

To examine how motor history affects temporal changes of brain activity, we attempted to visualize the functional connectivity reflected in the time-varying phase synchronization using our proposed method. Clustering results for the mental hand rotation task are shown in Figures 4, 5. Figure 4 shows the clustering results of the switch trial for each laterality condition in the mental hand rotation task [Figures 4A,C: left hand (switch)/Figures 4B,D: right hand (switch)]. Figure 5 shows the clustering result of the repeat trial for each laterality condition in the mental hand rotation task [Figures 5A,C: left hand (repeat)/Figures 5B,D: right hand (repeat)].

Figures 4, 5 only show clusters with cluster-averaged z-wPLIs that have a significantly positive amplitude (*p* < 0.05 with FDR correction). Note that the order of the cluster numbers in the results (Figures 4, 5) were determined in each trial condition (left: switch, right: switch, left: repeat, right: repeat) via hierarchical clustering. The effect of multiple comparisons was corrected using the FDR method.

Clustering analysis revealed a greater number of clusters in the switch trials compared to the repeat trials. We found similar clusters for both trial types (switch and repeat) with connectivity patterns that included multiple brain areas (left: switch, cluster 1, Figure 4A/right: switch, cluster 2, Figure 4B/left: repeat, cluster 2, Figure 5A/right: repeat, cluster 1, Figure 5B). These common clusters showed substantial negative changes in the cluster-averaged z-wPLIs around 0.5 s after task onset.

By focusing on the temporal changes of the cluster-averaged z-wPLIs (Figures 4C,D, 5C,D), we found that some clusters exhibited a tendency for the cluster-averaged z-wPLI values to increase in relation to the timing of the response (*p* < 0.05 with FDR correction, see the response-locked average values in the lower panels of Figures 4C,D, 5C,D). For instance, on switch trials, the clusters exhibiting a tendency for the cluster-averaged z-wPLIs to increase in relation to the timing of the response, showed functional connectivity, including the channels corresponding to the motor area (C3, Cz, and C4) (left: switch, cluster 1, 3, and 4 in Figure 4A/right: switch, cluster 1, 2, and 3 in Figure 4B). In addition, on repeat trials, we found that some clusters exhibited a similar tendency, and similar functional connectivity (left: repeat, cluster 1 and 2 in Figure 5A/right: repeat, cluster 1 and 3 in Figure 5B). Given that the clusters exhibiting the response-related tendency were detected regardless of the trial condition, we propose that the tendency of these clusters to exhibit increased z-wPLIs in relation to the timing of the response might reflect brain activity during the foot motor execution. On repeat trials (Figures 5A,B), the clusters, other than those corresponding to the foot motor execution, showed a tendency for the cluster averaged z-wPLIs to dramatically decrease around 0.5 s after task onset (left: repeat, cluster 4 / right: repeat, cluster 2 and 4).

In contrast to the repeat trials, switch trials involved specific clusters, including channel pairs between Pz and P4 (left: switch, cluster 8; right: switch, cluster 7), which exhibited different tendencies from the clusters in the repeat trials. The presence of switch-specific clusters indicates that the amplitude of the cluster-averaged z-wPLIs significantly increased (*p* < 0.05 with FDR correction) until the subject made a response (lower panels of Figures 4C,D), regardless of the selected hand laterality.

This tendency was observed not only for the stimulus-locked averaged values (shown in the upper panels of Figures 4C,D), but also for the response-locked values (lower panels of Figures 4C,D). The response-locked averaged wPLIs indicated that the positive amplitude appeared earlier relative to the response, and this significant increase was observed only on the switch trials.

### 3.3. Similarity analysis

To further examine whether this switch-specific activity only occurred during the mental hand rotation task, we evaluated the cluster-by-cluster similarity between alpha band connectivity patterns during switch trials for both task types (mental hand rotation task and command-to-response task) using cosine similarity (Mars et al., 2016). We then considered two conditions for the analysis: (1) similarity between two the laterality conditions (mental rotation task: left hand vs. right hand/command-to-response task: left angle bracket vs. right angle bracket) for each task on the switch trials, and (2) similarity between two tasks with the same laterality condition (left hand vs. left angle bracket/right hand vs. right angle bracket).

**Figure 6:**
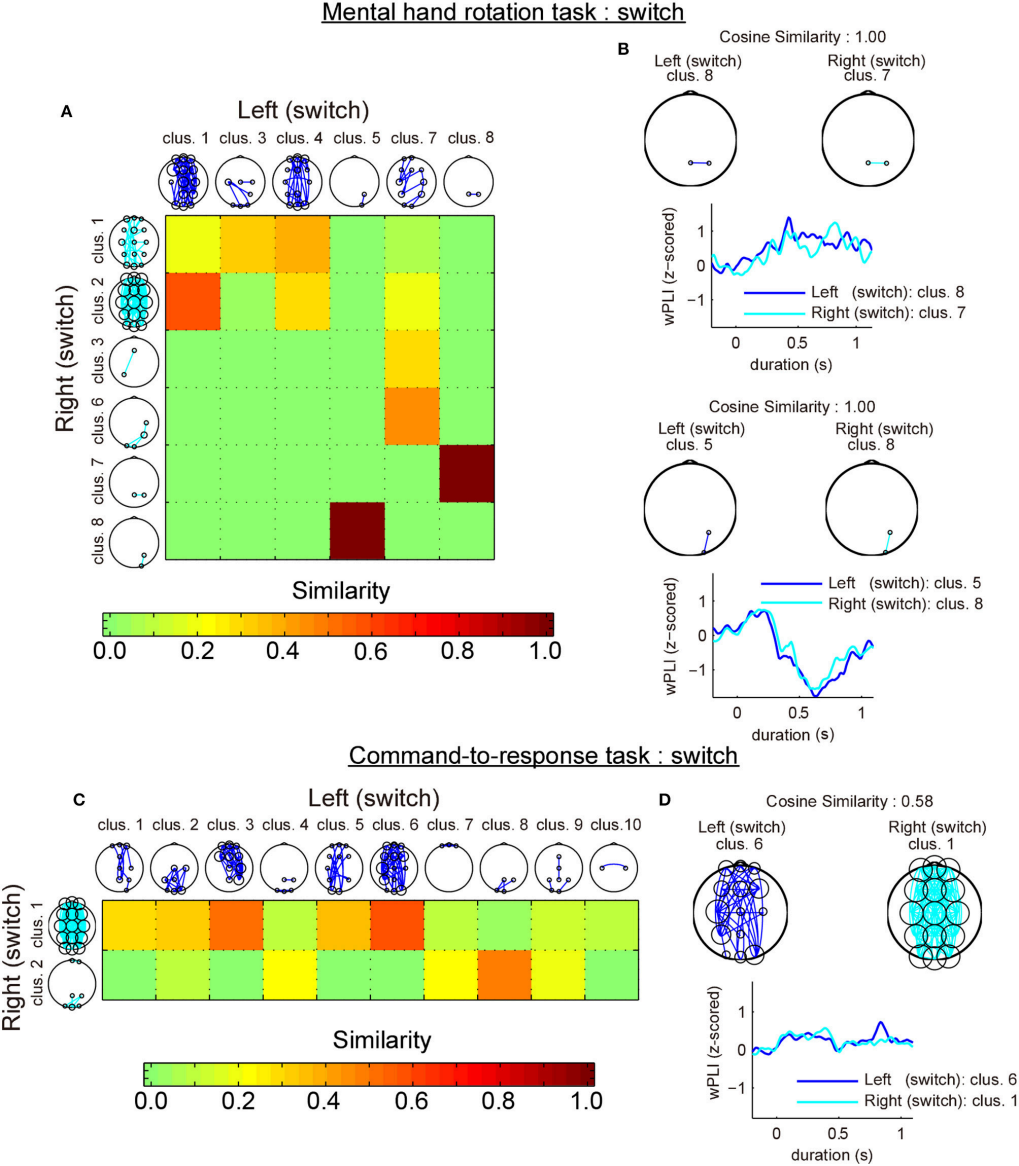
Comparison of the cluster-by-cluster similarity of the connectivity patterns (switch trials). **(A)** A matrix indicating the cluster-by-cluster similarity between left and right hand in the mental hand rotation task for the switch trials. **(B)** Pairs of most similar clusters between the two laterality conditions in the mental hand rotation task for switch trials and comparison of the temporal responses. **(C,D)** These also show the results of the cluster-by-cluster similarity analysis of the connectivity patterns in the command-to-response task for the switch trials.

**Figure 7:**
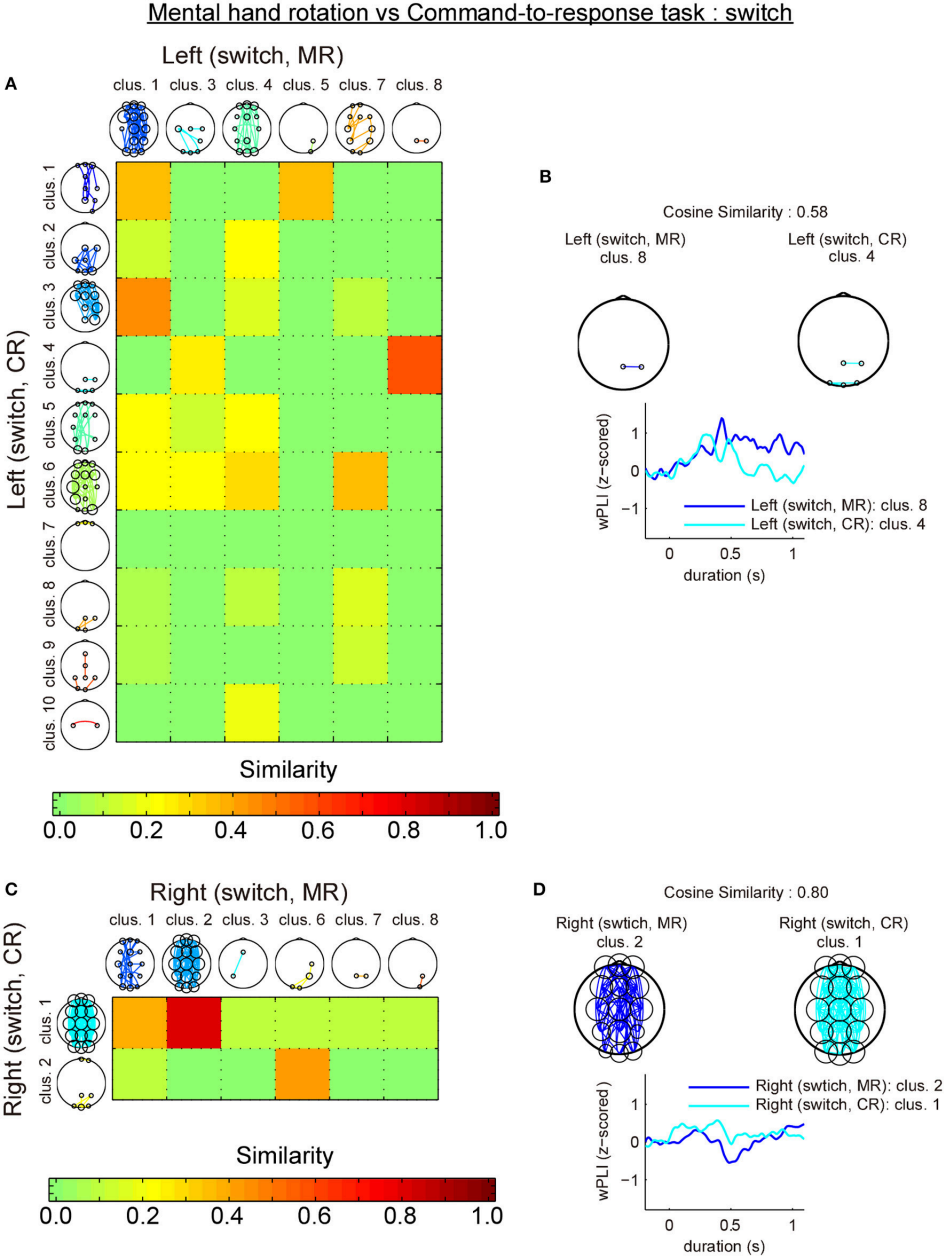
Comparison of the cluster-by-cluster similarity of the connectivity patterns (switch trials; mental hand rotation task vs. command-to-response task). **(A)** A matrix indicating the cluster-by-cluster similarity between the mental hand rotation and command-to-response task for the left condition in the switch trials. **(B)** Pairs of most similar clusters between the two tasks for the left conditions in the switch trials and comparison of the temporal responses. **(C,D)** These also show the results of the cluster-by-cluster similarity analysis of the connectivity patterns between the two tasks for the right condition in the switch trials. MR and CR indicate the mental hand rotation and command-to-response task, respectively.

The first analysis revealed that most similar cluster-pairs differed depending on the task. Figure 6A shows the cluster-by-cluster similarity between left and right hands in the mental hand rotation task for switch trials. Figure 6C shows the cluster-by-cluster similarity between left and right brackets in the command-to-response task for switch trials. In these two figures, the color in each element of the similarity matrix indicates the cluster-by-cluster similarity value, which is calculated by the cosine similarity. If the elements in this matrix are red, this indicates a similarity value of 1.0 (i.e., perfectly similar). Thus, the color of each element of this matrix indicates the extent of similarity of the related cluster pairs in comparison to the functional connectivity pattern in each cluster pair. Figure 6B shows the most similar cluster pairs and a comparison of temporal changes in z-wPLIs for each cluster pair in the mental hand rotation task for switch trials. Also, Figure 6D shows the most similar cluster pairs and a comparison of temporal changes in z-wPLIs for each cluster pair in the command-to-response task for the switch trials. As shown in Figure 6B, the cluster including the functional connectivity between channels Pz and P4 was selected as the most similar cluster in the mental hand rotation task during the switch trials. However, this cluster was not selected in the command-to-response task during switch trials (Figure 6D). In the second analysis, similarity analysis between the two tasks with the same laterality conditions of the presented stimuli revealed that the switch-specific cluster including connections between Pz and P4 in the mental rotation task also appeared in the command-to-response task during switch trials in which the left angle brackets were presented (Figures 7A,B). These results suggest the existence of a switch-specific cluster, regardless of the laterality of the selected hand and task. However, this cluster was not activated in switch trial in which right angle brackets were presented (Figures 7C,D).

## 4. Discussion

We applied a clustering-based analysis to multi-channel EEG signals recorded during the mental hand rotation and command-to-response tasks. The results revealed that for both tasks the extent of phase synchronization between parietal areas (Pz and P4) in the alpha-band of the EEG was altered by the history of the selected actions. These findings indicate that the observed phase synchronization between the parietal areas reflects brain activity evoked by action switching regardless of task paradigm, rather than cognitive processes that are specifically engaged during the mental hand rotation task. In previous studies of neural correlates (Helmich et al., 2009), results were only reported for the mental rotation task. Therefore, it remained unclear whether history dependency in action switching differs between the mental hand rotation task and general action switching tasks. By comparing the mental hand rotation task with the command-to-response task, the current findings suggest that common neural mechanisms of action switching underlying these two different tasks may induce modulation in the alpha-power phase synchronization in parietal areas, which might reflect the history-dependence of action selection in the brain.

Although we demonstrated the common effects of action switching on EEG signals, the current study involved several constraints that limit the generalizability of our findings as a neural signature of action switching. For example, in the command-to-response task, the switching-specific phase synchronization appeared only when left angle brackets were presented, whereas in the mental rotation task, it appeared in response to both right and left-hand stimuli. We believe that the lack of inter-parietal phase synchronization during right angle brackets stimuli in the command-to-response task may be due to different activity patterns in dominant and non-dominant effectors (foot). In our study, all participating subjects were right foot dominant. It is well known that simple movements with dominant effectors (hand or foot) are mainly associated with increase of activity in the contralateral sensorimotor areas, while both hemispheres are activated during non-dominant effector movements (Haaland et al., 2004). In addition, a previous study (Martin et al., 2011) suggested that the extent of this asymmetrical activity pattern in frontoparietal sensorimotor areas is larger for right-handers compared to that for left-handers during motor planning. In some cases, this strong asymmetric pattern could mask other brain activity and prevent its detection (Agnew and Wise, 2008). Considering these findings, overall activity in the ipsilateral (right) hemisphere might be small or suppressed during dominant foot movements (i.e., right angle brackets stimuli and right foot movements), and thus, it could be difficult to detect switching-specific phase synchronization. In contrast, this might be not the case for the mental hand rotation task, which requires subjects to compare the shape of a given visual stimulus with a mentally rotated hand before they can decide whether the given visual stimulus is a right or left hand. On the other hand, the neural processes underlying the command-to-response task are straightforward, and do not require such a comparison. Such a straightforward process underlying the command-to-response task might strongly affect the handedness-dependent effect of brain activity, relative to the mental hand rotation task, because both the contralateral and ipsilateral hemispheres are engaged and the extent of asymmetry is reduced in complex movements or tasks (Haaland et al., 2004). Thus, asymmetrical brain activity during simple dominant effector movement and differences in the computational process of decision-making between two tasks could be a factor explaining the observed phase synchronization. However, the fact that all subjects who participated in our study were right foot dominant indicates the need to further investigate the existence of hand/foot-dominance effect for switch-specific phase synchronization between parietal areas. This requirement of further studies illustrates the limitation of this study in generalizing the above discussion.

The differences of visual stimuli between the mental rotation and command-to-response tasks in the current study should also be considered. The mental hand rotation task involves mental simulation of a hand-rotating movement, as an additional process, as well as more complex sensory processing of the visual stimuli, relative to the command-to-response task. Thus, our findings could not eliminate the effects of the differences in the visual stimuli or mental simulation between the two tasks. To address this issue, we conducted an additional analysis, examining the effect of the visual stimuli and the mental simulation in each task without interaction with the motor history. In this additional analysis, we applied the same functional connectivity analysis to the pooled data from all stimuli and trial conditions for each task. The results revealed inter-occipital, occipito-parietal, and occipito-frontal connectivity (see Figures S11, S12C in the Supplementary Materials) as common functional connectivity for visual processing (mental hand rotation task: clusters 4 and 5 in Figure S11A/command-to-response task: clusters 1 and 2 in Figure S11B). In the results of this additional analysis for the mental hand rotation task, we observed additional clusters, beyond the common functional connectivity mentioned above between the two tasks (Figure S11A). For example, a cluster including the occipito-parietal region was observed in the mental hand rotation task (cluster 7 in Figure S11A), which indicates a tendency for the stimulus-locked averaged z-wPLI in this cluster to increase relative to the timing of task onset. Therefore, cluster 7 in Figure S11A is likely to reflect an effect of the differences in visual stimuli between the tasks or mental simulation of hands. Other clusters in the mental hand rotation task (e.g., clusters 1, 2, and 9 in Figure S11A) are likely to reflect an effect of the motor execution to their own response, because such clusters suggest that the response-locked average z-wPLI in such clusters increased relative to the timing of the response (see the lower panel in Figure S11C). However, as we mentioned above, this additional analysis did not identify the cluster containing the phase synchronization between Pz and P4. Given the points mentioned above, the findings of this additional analysis suggest that the switching-specific synchronization we observed in the parietal areas was not a direct effect of the difference in visual stimuli or mental simulation itself, but was more likely to be generated by the history of action selection. Thus, our results support the concept that the history-dependent interaction during the mental hand rotation task shares neural mechanisms with other action switching tasks. Of course, we should keep in mind that the additional analysis may not perfectly distinguish the effects of additional processing during the mental rotation task from neural activity underlying action switching, because there are several differences between the two tasks. Comparison with data from other switching tasks in future work may provide additional support for this claim.

How can we generalize our findings beyond action selection tasks? As a related finding, we consider the relationship between response-switching and response inhibition process, as suggested by Kenner et al. (2010). In their study, using a task combining a Go-No-Go component and a response switching component, Kenner et al. (2010) found that the neural contribution of some bran regions, including the frontal area, supplementary motor area, and parietal area, underlying response inhibition by stop-signals is shared with the mechanisms underlying task switching, by comparing the neural activity corresponding to response switching with that of response inhibition. The contribution of inhibitory processes to the response priming underlying task selection is also suggested by a previous study involving the Simon task (Treccani et al., 2017). Combining these findings with the current data, we speculate that the observed switching-specific inter-parietal alpha phase synchronization contributes to the inhibition of neural representations of previously selected actions, and that this function of the observed phase synchronization is shared between action selection and cognitive switching tasks. However, although Kenner et al. (2010) have suggested contributions of the frontal area as well as the parietal area to task selection and response inhibition, the current study observed alpha-power phase synchronization only in the parietal areas for switching-specific activity. The reason for the absence of the frontal area in action switching remains an open question. As a possible reason, we might consider the findings of a cognitive study involving task switching (Philipp et al., 2013), which mentioned that the extent of the contribution of the parietal and frontal cortices depended on the type of switching in the task. For example, according to this study, whereas the parietal area predominantly contributes to switching the response modality (e.g., hand or foot response), the frontal area predominantly contributes to switching the stimulus category (e.g., color vs. form). Considering these facts, since the mental hand rotation and command-to-response task that were used in the current study predominantly involved the effect of the history of response modality, we believe that inter-parietal phase synchronization is the brain activity specifically underlying action switching. However, as mentioned above, we should further consider the effect of motor dominance (i.e., handedness) underlying action switching to generalize the relationship between our findings and neural mechanism underlying the response inhibition.

In summary, we suggest that the history-dependent interaction proposed by Helmich et al. (2009) might reflect a common neural activity underlying action switching, rather than a mechanism that is specialized for the mental rotation task.

## 5. Conclusion

In the current study, we have provided experimental evidence that alpha-power inter-parietal synchronization is enhanced only when the selected action is switched from a previous action to a different action. These findings suggest that alpha-power inter-parietal synchronization is engaged as a form of switching-specific functional connectivity, and that switching-related activity exists independently of the task paradigm. This finding supports the conclusion that the history-dependent interaction suggested by Helmich et al. (2009) is a common neural activity underlying action switching, rather than one that is specialized for the mental rotation task. Thus, our results indicate that a history-dependent interaction during mental hand rotation task shares neural mechanisms with other general action switching tasks.

## Author contributions

HY, IN, JI, and YW conceived and designed the experiments. HY performed the experiments. HY analyzed the data. HY, IN, JI, and YW contributed reagents, materials, analysis tools. HY, IN, JI, and YW wrote the paper.

### Conflict of interest statement

The authors declare that the research was conducted in the absence of any commercial or financial relationships that could be construed as a potential conflict of interest.

## References

[B1] Agnew Z. Wise R. J. (2008). Separate areas for mirror responses and agency within the parietal operculum. J. Neurosci. 28, 12268–12273. 10.1523/JNEUROSCI.2836-08.2008 19020020 PMC6671715

[B2] Baayen R. H. Milin P. (2010). Analyzing reaction times. Int. J. Psychol. Res. 3, 12–28. 10.21500/20112084.807

[B3] Benjamini Y. Hochberg Y. (1995). Controlling the false discovery rate: a practical and powerful approach to multiple testing. J. R. Stat. Soc. 57, 289–300. 10.2307/2346101

[B4] Caliński T. Harabasz J. (1974). A dendrite method for cluster analysis. Commun. Stat. Theory Methods 3, 1–27. 10.1080/03610927408827101

[B5] Chen X. Bin G. Daly I. Gao X. (2013). Event-related desynchronization (erd) in the alpha band during a hand mental rotation task. Neurosci. Lett. 541, 238–242. 10.1016/j.neulet.2013.02.036 23458675

[B6] Cisek P. (2006). Integrated neural processes for defining potential actions and deciding between them: a computational model. J. Neurosci. 26, 9761–9770. 10.1523/JNEUROSCI.5605-05.2006 16988047 PMC6674435

[B7] Cohen M. X. (2015). Effects of time lag and frequency matching on phase-based connectivity. J. Neurosci. Methods 250, 137–146. 10.1016/j.jneumeth.2014.09.005 25234308

[B8] Cona G. Marino G. Semenza C. (2017). TMS of supplementary motor area (SMA) facilitates mental rotation performance: evidence for sequence processing in SMA. Neuroimage 146, 770–777. 10.1016/j.neuroimage.2016.10.032 27989840

[B9] Cooper L. A. Shepard R. N. (1975). Mental transformation in the identification of left and right hands. J. Exp. Psychol. Hum. Percept. Perform. 1:48 10.1037/0096-1523.1.1.48 1141835

[B10] de Lange F. P. Helmich R. C. Toni I. (2006). Posture influences motor imagery: an fmri study. Neuroimage 33, 609–617. 10.1016/j.neuroimage.2006.07.017 16959501

[B11] Haaland K. Y. Elsinger C. L. Mayer A. R. Durgerian S. Rao S. M. (2004). Motor sequence complexity and performing hand produce differential patterns of hemispheric lateralization. J. Cogn. Neurosci. 16, 621–636. 10.1162/089892904323057344 15165352

[B12] Hare T. A. Schultz W. Camerer C. F. O'Doherty J. P. Rangel A. (2011). Transformation of stimulus value signals into motor commands during simple choice. Proc. Natl. Acad. Sci. U.S.A. 108, 18120–18125. 10.1073/pnas.1109322108 22006321 PMC3207676

[B13] Helmich R. C. Aarts E. de Lange F. P. Bloem B. R. Toni I. (2009). Increased dependence of action selection on recent motor history in parkinson's disease. J. Neurosci. 29, 6105–6113. 10.1523/JNEUROSCI.0704-09.2009 19439588 PMC6665502

[B14] Horst A. C. Jongsma M. L. Janssen L. K. Lier R. Steenbergen B. (2012). Different mental rotation strategies reflected in the rotation related negativity. Psychophysiology 49, 566–573. 10.1111/j.1469-8986.2011.01322.x 22091978

[B15] Horst A. C. Lier R. Steenbergen B. (2013). Mental rotation strategies reflected in event-related (de) synchronization of alpha and mu power. Psychophysiology 50, 858–863. 10.1111/psyp.12076 23829384

[B16] Jeannerod M. (2001). Neural simulation of action: a unifying mechanism for motor cognition. Neuroimage 14, S103–S109. 10.1006/nimg.2001.0832 11373140

[B17] Karamzadeh N. Medvedev A. Azari A. Gandjbakhche A. Najafizadeh L. (2013). Capturing dynamic patterns of task-based functional connectivity with eeg. Neuroimage 66, 311–317. 10.1016/j.neuroimage.2012.10.032 23142654 PMC3609939

[B18] Kenner N. M. Mumford J. A. Hommer R. E. Skup M. Leibenluft E. Poldrack R. A. (2010). Inhibitory motor control in response stopping and response switching. J. Neurosci. 30, 8512–8518. 10.1523/JNEUROSCI.1096-10.2010 20573898 PMC2905623

[B19] Kent S. W. Wilson A. D. Plumb M. S. Williams J. H. Mon-Williams M. (2009). Immediate movement history influences reach-to-grasp action selection in children and adults. J. Motor Behav. 41, 10–15. 10.1080/00222895.2009.10125921 19073467

[B20] Koessler L. Maillard L. Benhadid A. Vignal J. P. Felblinger J. Vespignani H.. (2009). Automated cortical projection of eeg sensors: anatomical correlation via the international 10–10 system. Neuroimage 46, 64–72. 10.1016/j.neuroimage.2009.02.006 19233295

[B21] Kosslyn S. M. Digirolamo G. J. Thompson W. L. Alpert N. M. (1998). Mental rotation of objects versus hands: neural mechanisms revealed by positron emission tomography. Psychophysiology 35, 151–161. 10.1111/1469-8986.3520151 9529941

[B22] Lachaux J.-P. Rodriguez E. Martinerie J. Varela F. J.. (1999). Measuring phase synchrony in brain signals. Hum. Brain Mapp. 8, 194–208. 10.1002/(SICI)1097-0193(1999)8:4<194::AID-HBM4>3.0.CO;2-C 10619414 PMC6873296

[B23] Mars R. B. Verhagen L. Gladwin T. E. Neubert F.-X. Sallet J. Rushworth M. F. (2016). Comparing brains by matching connectivity profiles. Neurosci. Biobehav. Rev. 60, 90–97. 10.1016/j.neubiorev.2015.10.008 26627865 PMC6485474

[B24] Martin K. Jacobs S. Frey S. H. (2011). Handedness-dependent and-independent cerebral asymmetries in the anterior intraparietal sulcus and ventral premotor cortex during grasp planning. Neuroimage 57, 502–512. 10.1016/j.neuroimage.2011.04.036 21554968 PMC3114104

[B25] Matsuda T. Kitajo K. Yamaguchi Y. Komaki F. (2017). A point process modeling approach for investigating the effect of online brain activity on perceptual switching. Neuroimage 152, 50–59. 10.1016/j.neuroimage.2017.02.068 28242318

[B26] Matzke D. Love J. Wiecki T. V. Brown S. D. Logan G. D. Wagenmakers E.-J. (2013). Release the beests: Bayesian estimation of ex-gaussian stop-signal reaction time distributions. Front. Psychol. 4:918. 10.3389/fpsyg.2013.00918 24339819 PMC3857542

[B27] Meszlényi R. Peska L. Gál V. Vidnyánszky Z. Buza K. (2016). Classification of fmri data using dynamic time warping based functional connectivity analysis, in Signal Processing Conference (EUSIPCO), 2016 24th European (Budapest: IEEE), 245–249. 10.1109/EUSIPCO.2016.7760247

[B28] Müller M. (2007). Dynamic time warping, in Information Retrieval for Music and Motion (Heidelberg: Springer), 69–84.

[B29] Munzert J. Lorey B. Zentgraf K. (2009). Cognitive motor processes: the role of motor imagery in the study of motor representations. Brain Res. Rev. 60, 306–326. 10.1016/j.brainresrev.2008.12.024 19167426

[B30] Osuagwu B. A. Vuckovic A. (2014). Similarities between explicit and implicit motor imagery in mental rotation of hands: an eeg study. Neuropsychologia. 65, 197–210. 10.1016/j.neuropsychologia.2014.10.029 25446966

[B31] Philipp A. M. Weidner R. Koch I. Fink G. R. (2013). Differential roles of inferior frontal and inferior parietal cortex in task switching: evidence from stimulus-categorization switching and response-modality switching. Hum. Brain Mapp. 34, 1910–1920. 10.1002/hbm.22036 22438215 PMC6870067

[B32] Podzebenko K. Egan G. F. Watson J. D. (2002). Widespread dorsal stream activation during a parametric mental rotation task, revealed with functional magnetic resonance imaging. Neuroimage 15, 547–558. 10.1006/nimg.2001.0999 11848697

[B33] Sauseng P. Klimesch W. (2008). What does phase information of oscillatory brain activity tell us about cognitive processes? Neurosci. Biobehav. Rev. 32, 1001–1013. 10.1016/j.neubiorev.2008.03.014 18499256

[B34] Shimaoka D. Kitajo K. Kaneko K. Yamaguchi Y. (2010). Transient process of cortical activity during necker cube perception: from local clusters to global synchrony. Nonlinear Biomed. Phys. 4:S7. 10.1186/1753-4631-4-S1-S7 20522268 PMC2880804

[B35] ter Horst A. C. van Lier R. Steenbergen B. (2010). Mental rotation task of hands: differential influence number of rotational axes. Exp. Brain Res. 203, 347–354. 10.1007/s00221-010-2235-1 20376435 PMC2871105

[B36] Thayer Z. C. Johnson B. W. (2006). Cerebral processes during visuo-motor imagery of hands. Psychophysiology 43, 401–412. 10.1111/j.1469-8986.2006.00404.x 16916437

[B37] Tosoni A. Corbetta M. Calluso C. Committeri G. Pezzulo G. Romani G.. (2014). Decision and action planning signals in human posterior parietal cortex during delayed perceptual choices. Eur. J. Neurosci. 39, 1370–1383. 10.1111/ejn.12511 24612482

[B38] Treccani B. Cona G. Milanese N. Umiltà C. (2017). Sequential modulation of (bottom-up) response activation and inhibition in a response conflict task: a single-pulse transcranial magnetic stimulation study. Psychol. Res. 1–16. 10.1007/s00426-017-0863-9 28393259

[B39] Varela F. Lachaux J.-P. Rodriguez E. Martinerie J. (2001). The brainweb: phase synchronization and large-scale integration. Nat. Rev. Neurosci. 2, 229–239. 10.1038/35067550 11283746

[B40] Vinck M. Oostenveld R. Van Wingerden M. Battaglia F. Pennartz C. M. (2011). An improved index of phase-synchronization for electrophysiological data in the presence of volume-conduction, noise and sample-size bias. Neuroimage 55, 1548–1565. 10.1016/j.neuroimage.2011.01.055 21276857

[B41] Zacks J. M. (2008). Neuroimaging studies of mental rotation: a meta-analysis and review. J. Cogn. Neurosci. 20, 1–19. 10.1162/jocn.2008.20013 17919082

