# Supplementary material for: Alpha Phase Synchronization of Parietal Areas Reflects Switch-Specific Activity During Mental Rotation: An EEG Study
Source: Front Hum Neurosci. 2018 Jun 21;12:259. doi: 10.3389/fnhum.2018.00259 (PMC6021508; doi:10.3389/fnhum.2018.00259)
Supplement: Supplementary file 1 [file Data_Sheet_1.pdf]

Supplementary Material: Alpha Phase Synchronization of Parietal Areas Reflects Switch-Specific Activity During Mental Rotation: an EEG study

1 SUPPLEMENTARY METHODS

1.1 Dynamic time warping

In our proposed time-series data clustering procedure, we applied the dynamic time-warping (DTW) method(Sakoe and Chiba, 1978; Müller, 2007; Meszlényi et al., 2016; Karamzadeh et al., 2013) to optimally quantify the temporal similarity between two different time-series data sets. DTW is an algorithm

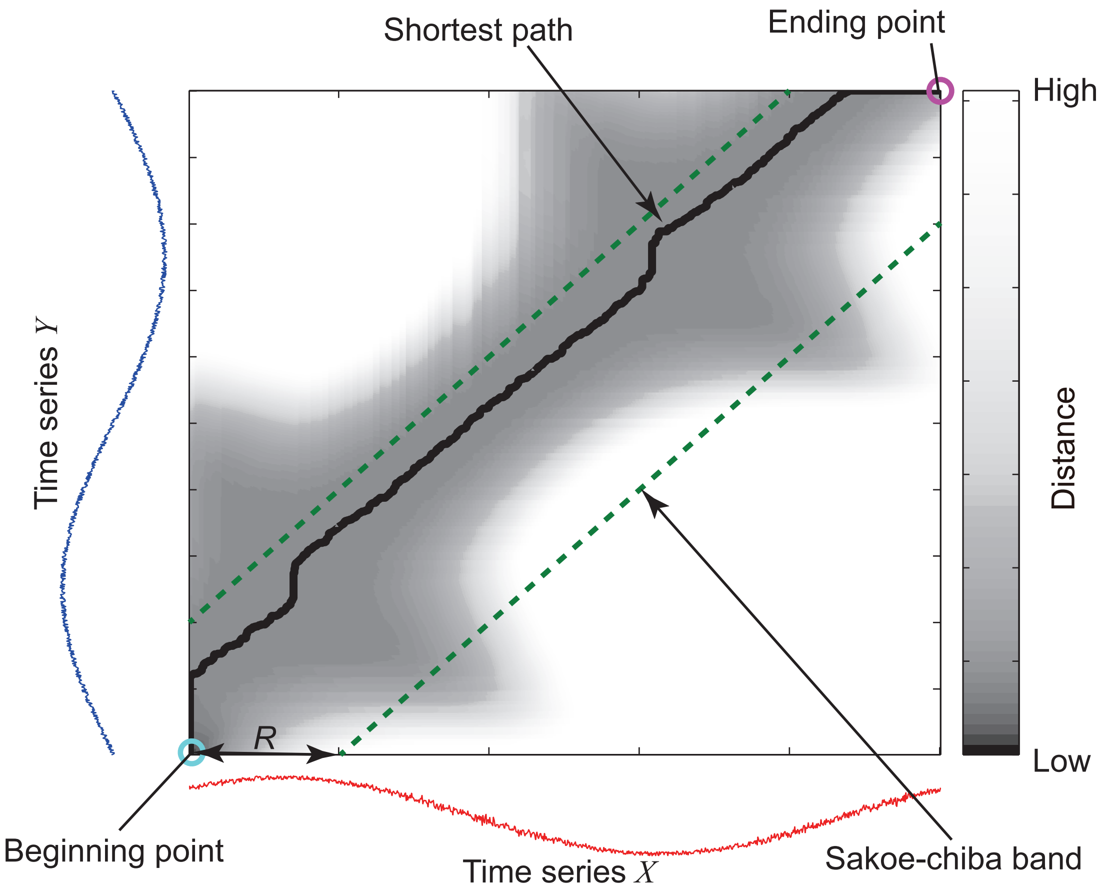

Figure S1: **Overview of DTW algorithm.**

for evaluating optimal matching between two different time series data sets X and Y of length L. This algorithm operates as follows:

1. Calculate the distance between each point in time series X and all the points in time series Y with Euclidean distance representing equation (S1).
   This calculation results in the $L \times L$ square matrix $c_p(X,Y)$.
   Here, the bottom-left and top-right corners of the matrix represent the distance between their beginning and end points (Fig. S1).

$$c_p(X,Y) = (x_i - y_i)^2, \qquad (i = 1, 2, ..., L) \tag{S1}$$

   where $c_p(X,Y)$ indicates the $L \times L$ cost function, which is constructed with all pairwise distances between $X = (x_1, x_2, ..., x_L)$ and $Y(y_1, y_2, ..., y_L)$, N is the total number of samples.
2. Find the shortest path n_i and m_i toward from bottom left corner to top right corner so that the total distance of the selected path is the smallest, using the resulting matrix in Step 1.
3. The total distance of the estimated shortest path in Step 2 is selected as the optimal distance $DTW(X,Y)$ indicating temporal similarity between X and Y.

$$DTW(X,Y) = \sum_{i=1}^{N} c_{p^*}(x_{n_i}, y_{m_i}) = \min\{c_p(x_{n_i}, y_{m_i})\} \tag{S2}$$

   where x_{n_i} and y_{m_i} are the elements of the cost function c_p associated with the index i of the selected path.

In Step 2, the shortest path is selected with the local constraint as follows:

$$\min\{c_p(x_{n_i-1}, y_{m_i}), c_p(x_{n_i-1}, y_{m_i-1}), c_p(x_{n_i}, y_{m_i-1})\}. \tag{S3}$$

Further, we applied the Sakoe-Chiba band(Sakoe and Chiba, 1978) as the global constraints for the selection of the shortest path. Considering the Sakoe-Chiba band, each element of the shortest path in Step 2 was selected with the ranges from each diagonal element to R samples.

In the analysis, the DTW was applied for time course matching for group averaged z-wPLI, to construct the distance matrix. Moreover, the window size R of DTW was set as $R = 15$ samples, referring to the results of the simulation described in Section 2 below.

2 EVALUATION OF PROPOSED METHOD

To test the statistical validity of our proposed clustering method for phase synchronization data, we applied this method to simulation data for which the group labels for all data were explicitly known. For this simulation, we applied the Kuramoto phase oscillator model (Stam et al., 2007; Kuramoto, 1975) to construct the simulation signal.

2.1 Parameter setting of Kuramoto model

The Kuramoto model(Stam et al., 2007; Kuramoto, 1975) is represented as a differential equation, as follows:

$$\frac{\partial \theta_i(t)}{\partial t} = \omega_i(t) + \zeta_i + K \sum_{j}^{N} \sin(\theta_j(t) - \theta_i(t)), \quad i = 1...N, \quad t \geq 0 \tag{S4}$$

where ω_i is the eigenfrequency, θ_i is the phase angle, K is the coupling parameter (constant number), N is the number of oscillators, i and j indicates the channel indices. Moreover, we added two random noise parameters ζ_i to represent the fluctuation.

In the simulation, we generated two different 3-coupled phase oscillators $\theta_i(t)$ (2 types $\times 3$ signals = 6 phase signals) based on the parameter setup described below. The generated signals contained 4.6 s samples, with a sampling frequency of 1024 (total 4710 samples), and the sample relevant to the 2.3 s period from the first sample was considered to indicate task onset (0.0 s). To consider the effect by that the samples near the points of both ends of simulated signal are distorted by signal processing (e.g. filtering, inverse Fourier transform), we used data including the interval from -0.4 s to 1.5 s for the analysis. In addition, the 0.4 s period before task onset was used as the baseline interval for data correction (see the Material and Method section in the manuscript for details).

Here, we prepared two groups of simulated electroencephalography (EEG) data channels (channels 1-3 and channels 4-6). Parameter setting was applied for each group, as follows:

[Channel 1 to 3]

$K = 0.03$

$\omega_i(t) = 2\pi f$

$$f = \begin{cases} 30 & : 0.1 \le t \le 0.4(\mathrm{s}) \\ \mathcal{U}(0, 30) & : otherwise \end{cases}$$

$$\zeta_i = \begin{cases} 0 & : 0.1 \le t \le 0.4(\mathrm{s}) \\ \mathcal{N}(0, 30) & : otherwise \end{cases}$$

[Channel 4 to 6]

$K = 0.03$

$\omega_i(t) = 2\pi f$

$$f = \begin{cases} 30 & : 0.4 \le t \le 0.7(\mathrm{s}) \\ \mathcal{U}(0, 30) & : otherwise \end{cases}$$

$$\zeta_i = \begin{cases} 0 & : 0.4 \le t \le 0.7(\mathrm{s}) \\ \mathcal{N}(0, 30) & : otherwise \end{cases}$$

These settings were intended to construct the artificial signals including the characteristics of actual EEG signals that exhibit phase oscillatory activity between different regions reflected in the beta frequency band (around 30 Hz). Therefore, the eigenfrequency ω_i was adjusted so that the oscillators in each group were synchronized with a 30 Hz rhythm at specific time-periods (Channel 1 to 3: $0.1 \le t \le 0.4[\mathrm{s}]$ / Channel 4 to 6: $0.4 \le t \le 0.7[\mathrm{s}]$). In the other time-periods, the ω_i fluctuated by the randomized frequency f based on the uniform distribution from 0 to 30 Hz ($\mathcal{U}[0, 30]$).

2.2 Analysis procedure of simulation data

First, we solved differential equations of the Kuramoto model with two different 3-coupled phase oscillators based on the parameter setup described above. We applied inverse fast Fourier transform to the resulting phase $\theta_i(t)$ (2 types $\times 3$ signals = 6 phase signals) to transform the simulated signals from phase-domain to time-domain signals. The time-domain signals were evaluated with the following equations.

$$F(t) = \exp(j\theta_i(t)) \tag{S5}$$

$$f(x) = \frac{1}{N}\sum_{t=0}^{N-1} F(t)\exp(j\frac{2\pi tx}{N}), \tag{S6}$$

where $F(t)$ indicates the frequency-domain signals with time t, $f(x)$ indicates the time-domain signals with time sample x, j is an imaginary number, and N is number of samples. Next, applying the real part of the $f(x)$ as the template signals, we artificially generated the simulated EEG signals 30 times, as follows:

1. We took the real part of time-domain signals $f(x)$ (Eq. [S5]) as the template for simulated signals.
2. White noise was added for the template signals.
3. The simulated signals were repeatedly generated 30 times based on the procedure described in steps 1 and 2.
4. The time frequency spectrum and weighted phase-lag index (wPLI) were calculated (see the text in the main manuscript for details).
5. The z-scored value of wPLIs (z-wPLIs) was computed for each pair of simulated signals ($6 \times (6 - 1)/2 = 15$ pairs).
6. The time-course similarity in each z-wPLI was evaluated using the DTW algorithm to create a distance matrix as an input value for hierarchical clustering (15×15 matrix).
7. Hierarchical clustering (Shimaoka et al., 2010) was applied with the average linkage algorithm.
8. The number of clusters in the cluster tree was estimated, which was evaluated in step 7.
9. The cluster-averaged time-course of z-wPLIs was visualized for each identified cluster.

The cluster number for the simulation data was evaluated using the $Pseudo\,F$ index(Caliński and Harabasz, 1974) (see the main manuscript for details). This index describes the ratio of between-cluster variance to within-cluster variance. Higher values on this index indicate greater cluster separation. Moreover, the clustering results for the simulation data were tested with the same statistical procedures as those used for the actual EEG data.

In this evaluation, we expected that our proposed method would categorize these two different groups of simulated phase oscillatory signals without confusion, by considering the temporal similarity of phase synchronization for each signal.

2.3 Results of Simulation

First, we obtained the results of a phase synchronisation analysis for the simulation data by applying same procedures as for the actual EEG data (Fig. S2). In this figure, the colour indicates the temporal changes of z-wPLIs for all signal-pairs and each frequency band. The intervals shown in red represent timing where the amplitude of phase synchronisation was strongly increased compared with the rest intervals. This result suggested that all simulated signals detected the phase oscillatory tendency, defined by this simulation setting. All signal-pairs between signal 1 and 3 showed a tendency for the signals to be synchronised between the intervals from 0.1 s to 0.4 s compared with after task-onset. All signal-pairs from signal 4 to signal 6 showed a tendency for the signals to be synchronised between the intervals from 0.4 s to 0.7 s compared with after task-onset. Further, we applied the same procedure used for the actual EEG data to the z-wPLIs of the simulated data. We set the window size of the DTW as $R = 5$ samples. The results suggested that the clusters were correctly estimated according to the phase oscillatory property for each simulation signal (Fig. S3(b)). The temporal response of cluster-averaged z-wPLIs exhibited significant intervals only for clusters exhibiting the same phase oscillatory signals (Fig. S3(a)).

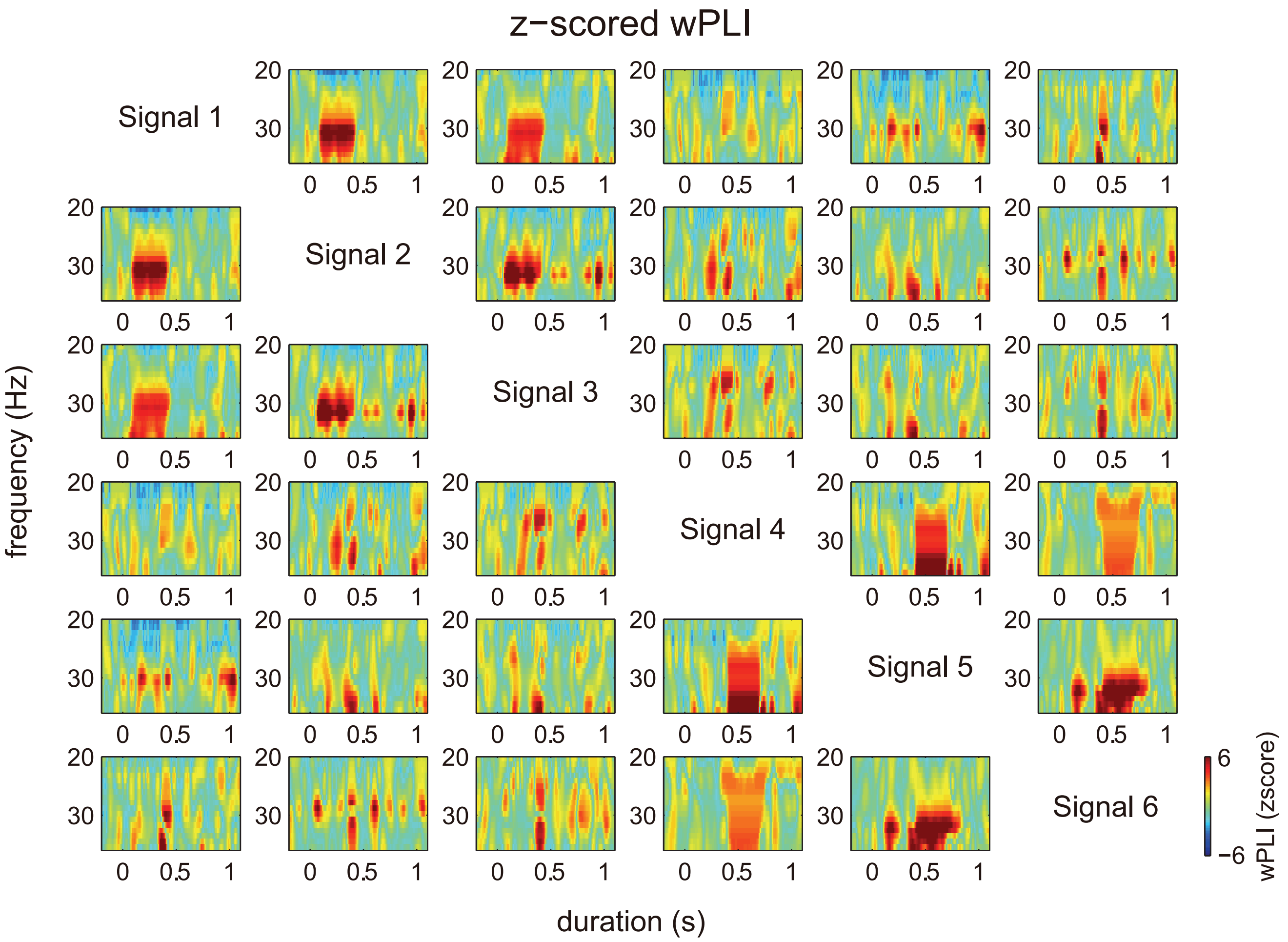

Figure S2: **z-scored wPLIs for simulation data.**

These findings suggest that our proposed method was able to correctly capture the temporal properties of phase synchronisation between measured signals.

2.3.1 Relationship between clustering results and window size $\boldsymbol{R}$ of the DTW

The results shown in Fig. S3 support the feasibility of our clustering method based on temporal similarity. However, the parameter-setting of the DTW was arbitrary. We then examined how the parameter R of the DTW affected the clustering results by comparing the determined number of clusters while the window size R changed from 1 to 50 samples.

Fig. S4 shows the effect of the parameter R in the DTW on the clustering results. The results revealed that the estimated number of clusters and Pseudo F index were stabilised as long as the R was below around 20 samples. However, these values suddenly changed as the window size exceeded 20 samples, and the $Pseudo\ F$ index declined in response to increasing the evaluated number of clusters. These results suggest that the efficiency of clustering decreased with oversized values for parameters R.

For this reason, in the analysis of actual EEG data, the size of the window parameter $R = 15$ was chosen to ensure that the selected values were sufficiently smaller than the boundary at which the tendency was drastically changed in the simulation.

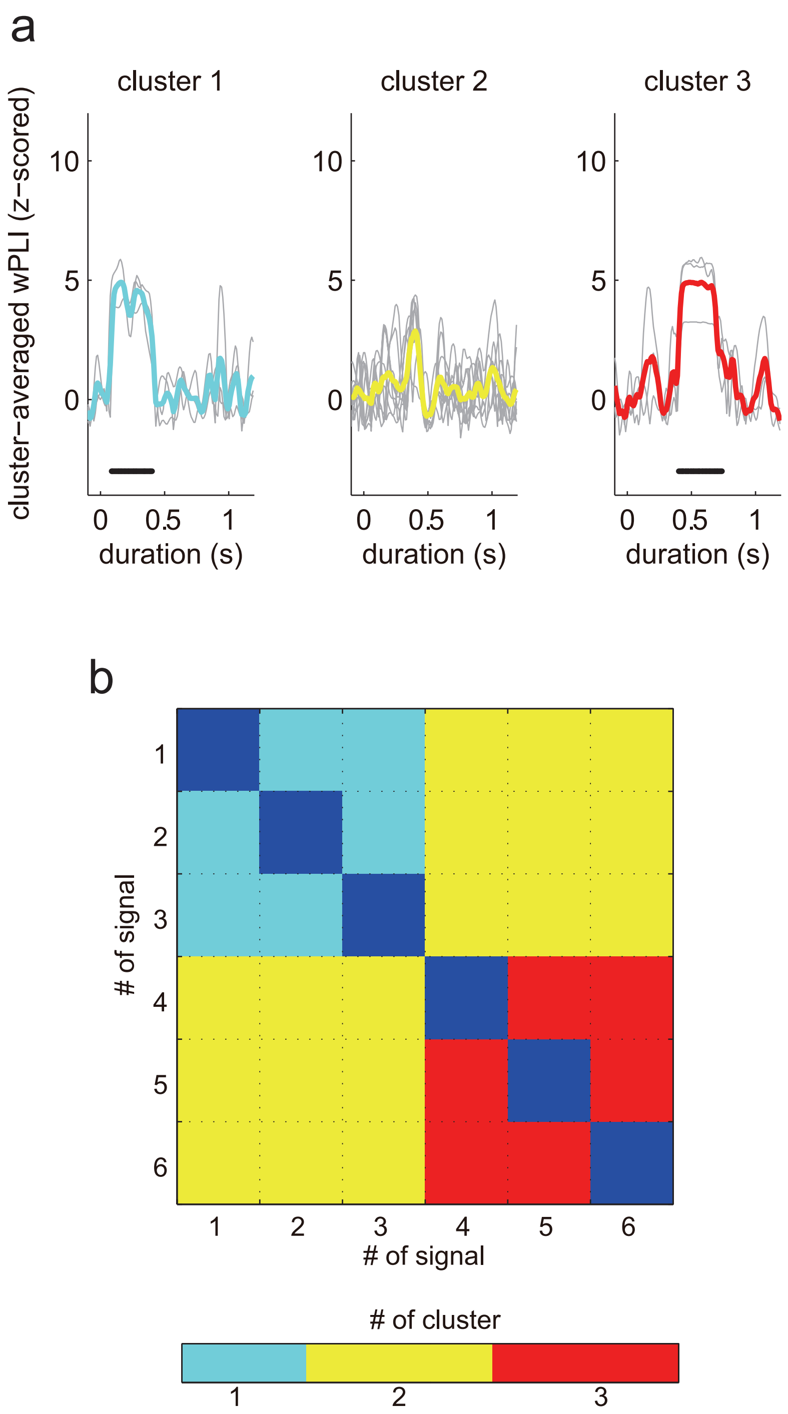

Figure S3: **Clustering results for the simulated data.** (a) The determined clusters and averaged value of z-scored wPLIs for each cluster. The asterisk indicates the significant interval of averaged values in the statistical test using same procedures as for the actual EEG data ($P < 0.05$ with FDR correction). (b) The cluster label index. This colour matrix indicates the cluster labels for each signal, with the colours associated with the number of clusters. The row and column index are associated with the number of signals.

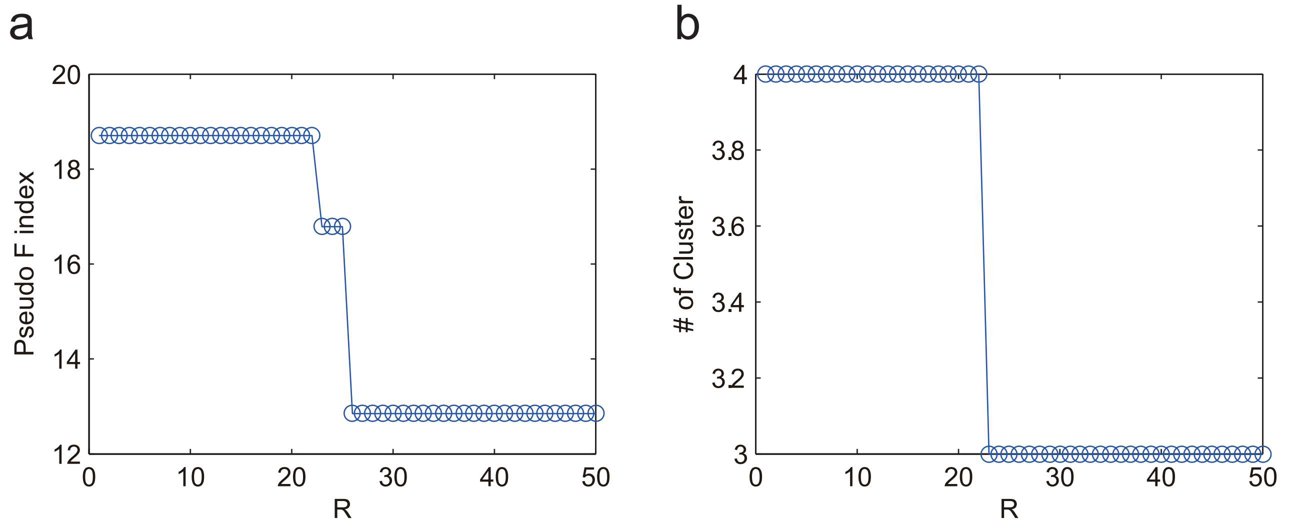

Figure S4: **Effect of clustering results with respect to the window size R of the DTW.** (a) The relationship between the Pseudo F index and R. (b) The relationship between the determined cluster number and R.

2.4 Software availability

In the interest of reproducibility, a software implementation of the above simulation and our proposed method of functional connectivity analysis as described in this work is available as a set of Matlab scripts. The codes can be found at the following link: `https://github.com/hiroshi-yokoyama/Yokoyama_et_al_front_hum_neurosci_2018.git`.

3 SUPPLEMENTARY RESULTS FOR BETA BAND

In this section, we show the clustering results of the other two frequency bands (Beta 1: 16-24 Hz / Beta 2: 28-36 Hz) in both the mental hand rotation task and command-to-response task. All results only show the clusters with significantly positive amplitude of cluster-averaged z-wPLIs ($\mathrm{P} < 0.05$ by statistical testing based on the surrogate method. The effect of multiple comparisons was corrected using the false discovery rate; FDR). We applied the same method of statistical testing used for the actual EEG data in the Alpha band (see Method for the details).

3.1 Mental hand rotation task

3.1.1 Mental hand rotation task : switch, Beta1

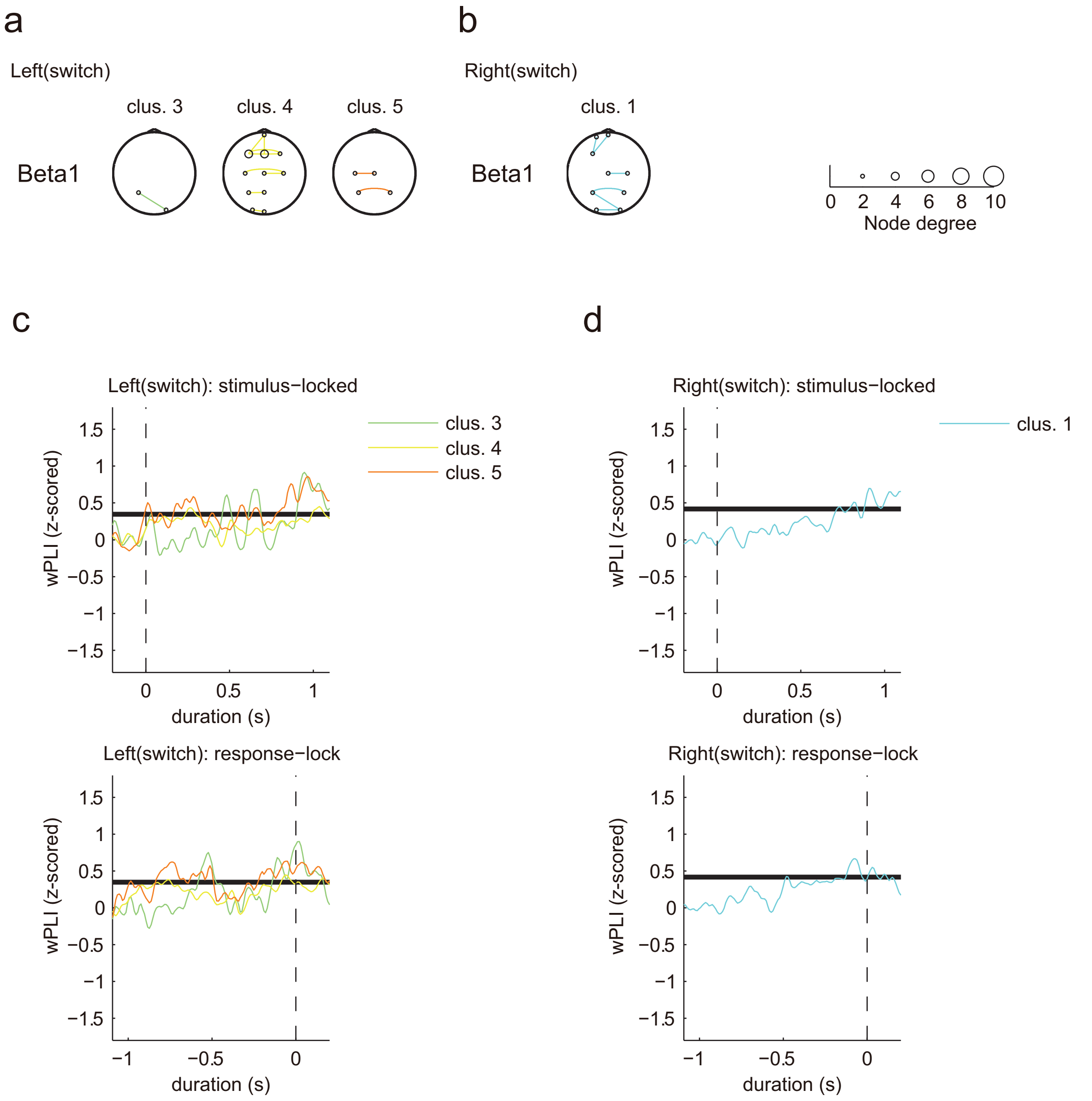

Figure S5: **Estimated cluster and functional connectivity (mental hand rotation task: switch trial, Beta1).** (a) Estimated significant clusters of functional connectivity for the left hand in switch trials. Three of five clusters were estimated as significant. (b) Estimated significant clusters of functional connectivity for the right hand in switch trials. Both clusters were estimated as significant. Marker size of electrodes for each topography corresponds to the node degree of connectivity. (c, d) Cluster-averaged z-scored wPLIs for each hand order in switch trials (upper panels: stimulus-locked average; lower panels: response-locked average). Bold black lines indicate a significant level of temporal changes of cluster-averaged phase-synchronisation values ($P < 0.05$ with FDR correction).

3.1.2 Mental hand rotation task : repeat, Beta1

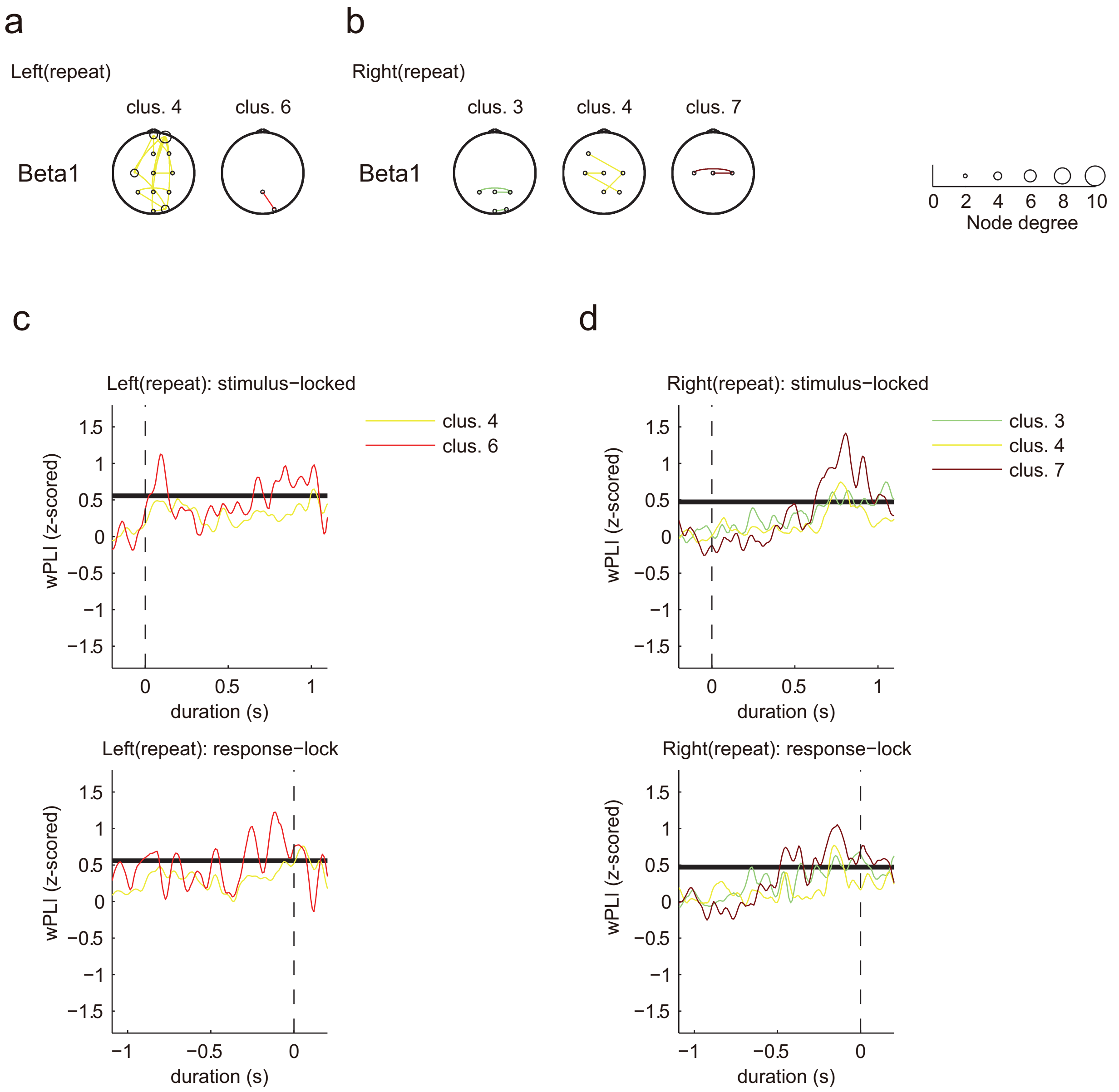

Figure S6: **Estimated clusters and functional connectivity (mental hand rotation task: repeat trial, Beta1).** (a) Estimated clusters of functional connectivity for the left hand in repeat trials. Two of six clusters were estimated as significant. (b) Estimated clusters of functional connectivity for the right hand in repeat trials. Three of seven clusters were estimated as significant. Marker size of electrodes for each topography corresponds to the node degree of connectivity. (c, d) Cluster-averaged z-scored wPLIs for each hand order in switch trials (upper panels: stimulus-locked average; lower panels: response-locked average). Bold black lines indicate a significant level of temporal changes of cluster-averaged phase-synchronisation values ($P < 0.05$ with FDR correction).

3.1.3 Mental hand rotation task : switch, Beta2

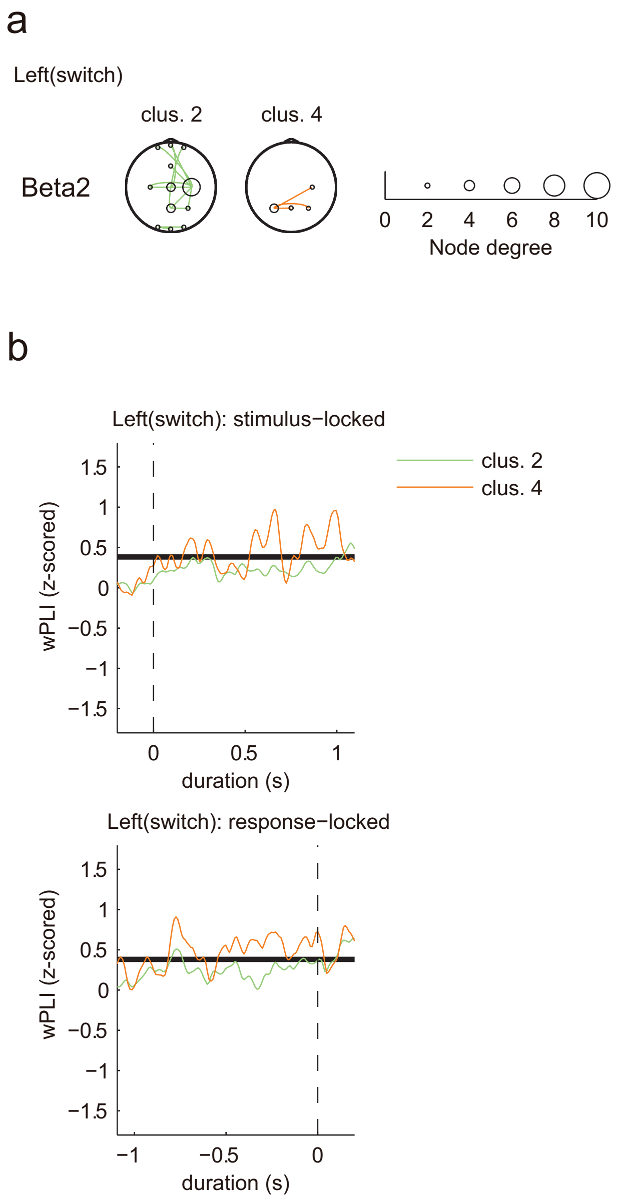

Figure S7: **Estimated cluster and functional connectivity (mental hand rotation task: switch trials, Beta2).** (a) Estimated clusters of functional connectivity for the left hand in switch trials. Two of four clusters were estimated as significant. Marker size of electrodes for each topography corresponds to the node degree of connectivity. (b) Cluster-averaged z-scored wPLIs in switch trials (upper panels: stimulus-locked average; lower panels: response-locked average). Bold black lines indicate a significant level of temporal changes of cluster-averaged phase-synchronisation values ($P < 0.05$ with FDR correction).

3.2 Command-to-response task

3.2.1 Command-to-response task : switch, Beta1

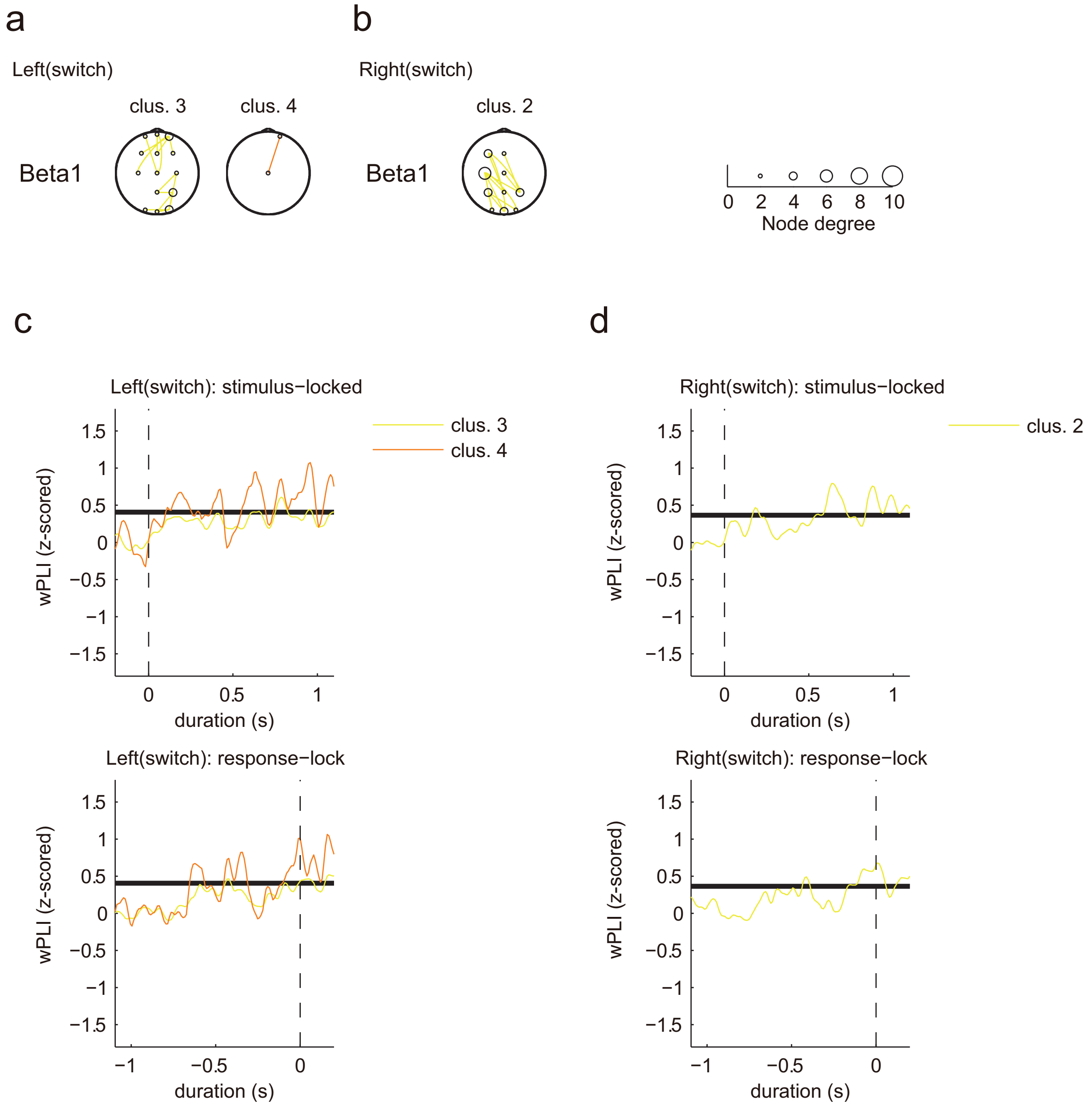

Figure S8: **Estimated clusters and functional connectivity (command-to-response task: switch trials, Beta1).** (a) Estimated clusters of functional connectivity for the left hand in switch trials. Two of four clusters were estimated as significant. (b) Estimated clusters of functional connectivity for the right hand in switch trials. One of two clusters was estimated as significant. Marker size of electrodes for each topography corresponds to the node degree of connectivity. (c, d) Cluster-averaged z-scored wPLIs for each hand order in switch trials (upper panels: stimulus-locked average; lower panels: response-locked average). Bold black lines indicate a significant level of temporal changes of cluster-averaged phase-synchronisation values ($P < 0.05$ with FDR correction).

3.2.2 Command-to-response task : repeat, Beta1

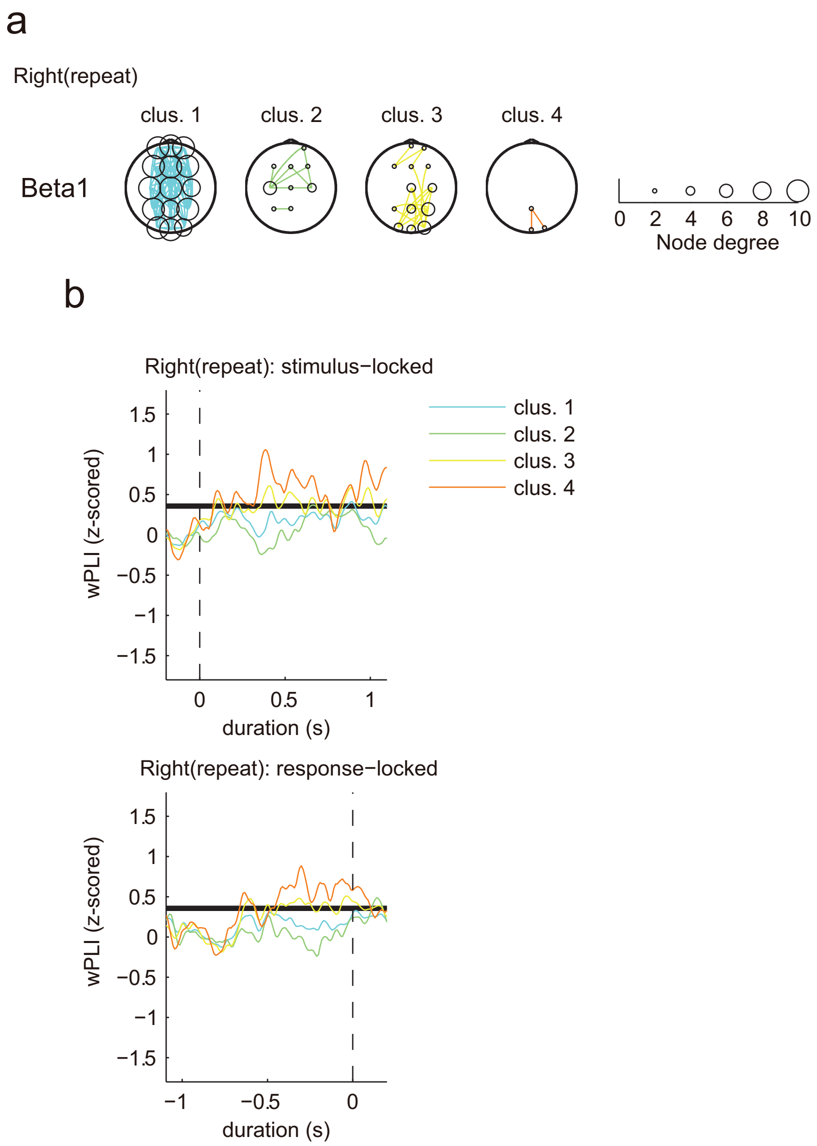

Figure S9: **Estimated clusters and functional connectivity (Command-to-response task: repeat trial, Beta1).** (a) Estimated clusters of functional connectivity for each hand order for the right hand in repeat trials. Marker size of electrodes for each topography corresponds to the node degree of connectivity. All four clusters were estimated as significant. (b) Cluster-averaged z-scored wPLIs (upper panels: stimulus-locked average; lower panels: response-locked average). Bold black lines indicate a significant level of temporal changes of cluster-averaged phase-synchronisation values ($P < 0.05$ with FDR correction).

3.2.3 Command-to-response task : switch, Beta2

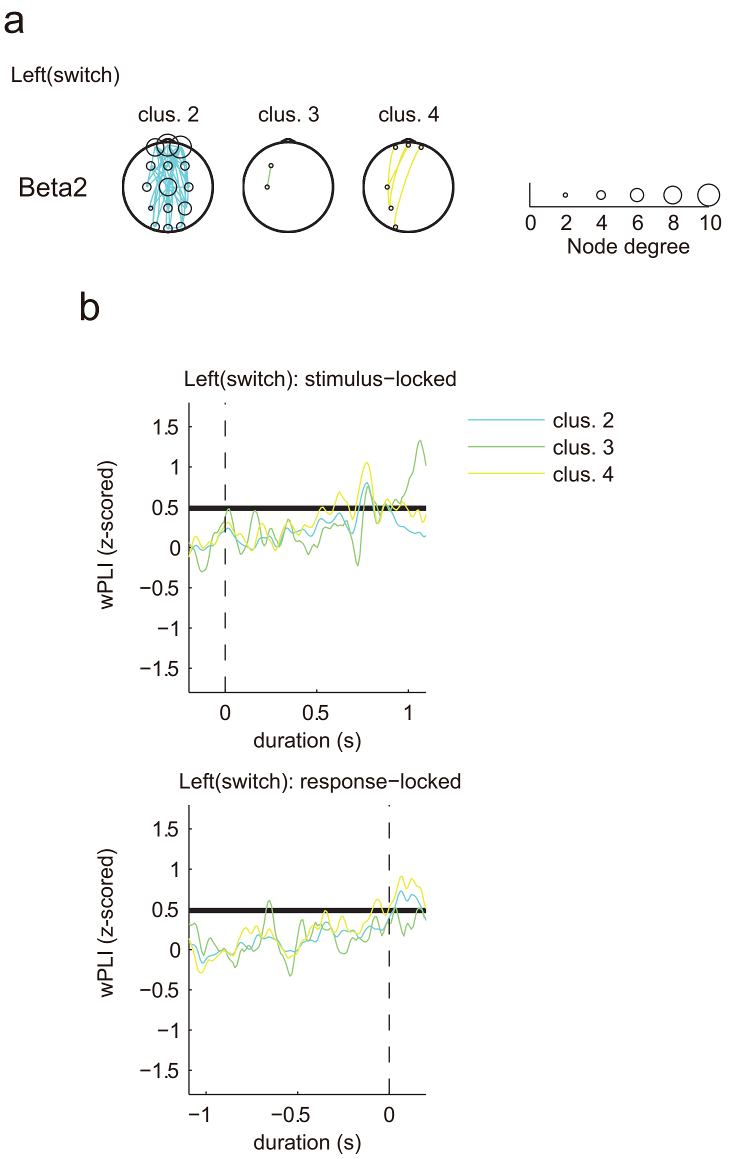

Figure S10: **Estimated clusters and functional connectivity (command-to-response task: switch trials, Beta2).** (a) Estimated clusters of functional connectivity for each hand order in the left hand in switch trials. Three of five clusters were estimated as significant. Marker size of electrodes for each topography corresponds to the node degree of connectivity. (b) Cluster-averaged z-scored wPLIs (upper panels: stimulus-locked average; lower panels: response-locked average). Bold black lines indicate a significant level of temporal changes of cluster-averaged phase-synchronisation values ($P < 0.05$ with FDR correction).

4 SUPPLEMENTARY ANALYSIS

4.1 Supplementary analysis procedure

Even though the two experiments which the visual stimuli were completely different were applied, main result of the paper could not provide the efficient explanation how the physical characteristics of visual stimuli depending on the task affects the time-varying phase oscillatory activity. To consider this issue, we have conducted additional analysis for functional connectivity evaluation.

4.1.1 Functional connectivity analysis

To specifically examine the task-dependent effects of the visual stimuli, an additional analysis was conducted for each task (mental hand rotation task and command-to-response task), using the following procedures.

1. Pre-processing and rejection of outlier trials were conducted using the same procedure described in the main manuscript
2. All EEG data were pooled to combine both laterality conditions and trial conditions for each task, for each subject
3. Z-scored wPLIs were calculated for each subject
4. Group-level functional connectivity was evaluated using the procedure described in the main manuscript

It should be noted that the EEG data corresponding to the excluded trials in the behavioral analysis were also removed from this supplementary analysis. The other procedures (such as pre-processing, calculation of z-wPLIs, functional connectivity analysis, and statistical testing of evaluated clusters) were applied using the method described in the main manuscript. This supplementary analysis was conducted on the EEG data for each task.

4.1.2 Similarity analysis

To evaluate the cluster-by-cluster similarity of connectivity patterns, we analyzed cosine similarity (Mars et al., 2016), examining the similarity in each pair of evaluated clusters in both tasks (mental rotation task: ALL trials vs command-to-response task: ALL trials).

4.2 Results of supplementary analysis

The results of the functional connectivity analysis are shown in Figure S11. In the mental hand rotation task, the analysis revealed that eight of 10 clusters were estimated to exhibit significant connectivity. In addition, in the command-to-response task, two clusters were estimated to exhibit significant connectivity. The analysis identified a similar cluster between both tasks, which included functional connectivity among regions corresponding to the occipital, parietal, and frontal cortices (mental hand rotation task; cluster 4 / command-to-response task; cluster 2). The averaged alpha band z-wPLI values of the regions exhibiting connectivity indicated an increase relative to the appearance of visual stimuli, which did not depend on the differences in visual stimuli between the mental hand rotation task and command-to-response task (see Figure S11C, D).

To examine this issue in more detail, we evaluated the similarity of the connectivity pattern in each pair of evaluated clusters in both tasks. This results are shown in Figure S12. Cluster 5 in the mental hand rotation task was identified as the most similar to cluster 1 in the command-to-response task (Figure S12B).

These two clusters exhibited a connectivity pattern that included multiple brain areas. A similar cluster was also observed in the results reported in the main manuscript (left: switch, cluster 1, Figure 4A / right: switch, cluster 2, Figure 4B / left: repeat, cluster 2, Figure 5A / right: repeat, cluster 1, Figure 5B).

The results revealed that cluster 4 in the mental hand rotation task was most similar to cluster 2 in the command-to-response task, which exhibited functional connectivity among the regions corresponding to the occipital, parietal, and frontal cortex. Several previous studies reported that alpha rhythm oscillation in multiple regions, including occipital, parietal and frontal cortex, play an important role in visual attention (Bressler et al., 2008; Romei et al., 2010; Bauer et al., 2012). Given that these two clusters (cluster 4 in the mental hand rotation task and cluster 2 in the command-to-response task) showed a tendency for the amplitude of cluster-averaged z-wPLIs to increase relative to the appearance of visual stimuli, independent of the difference in the visual stimuli between tasks. Such a functional connectivity pattern among clusters is consistent with physiological evidence.

Furthermore, in the mental hand rotation task, the results revealed an additional cluster including the occipito-parietal region, which was not observed in the other task condition (see cluster 7 in Figure S11). We consider that this additional cluster in the mental hand rotation task is likely to reflect an effect of the difference in visual stimuli between the tasks.

The additional analysis indicates that these clusters exhibited different functional connectivity patterns compared with the switch-related cluster reported in the main manuscript. Thus, the switch-related functional connectivity we observed, as reflected in the alpha power phase synchronization between parietal areas, appears likely to be independent of the effects of differences in the visual stimuli between tasks.

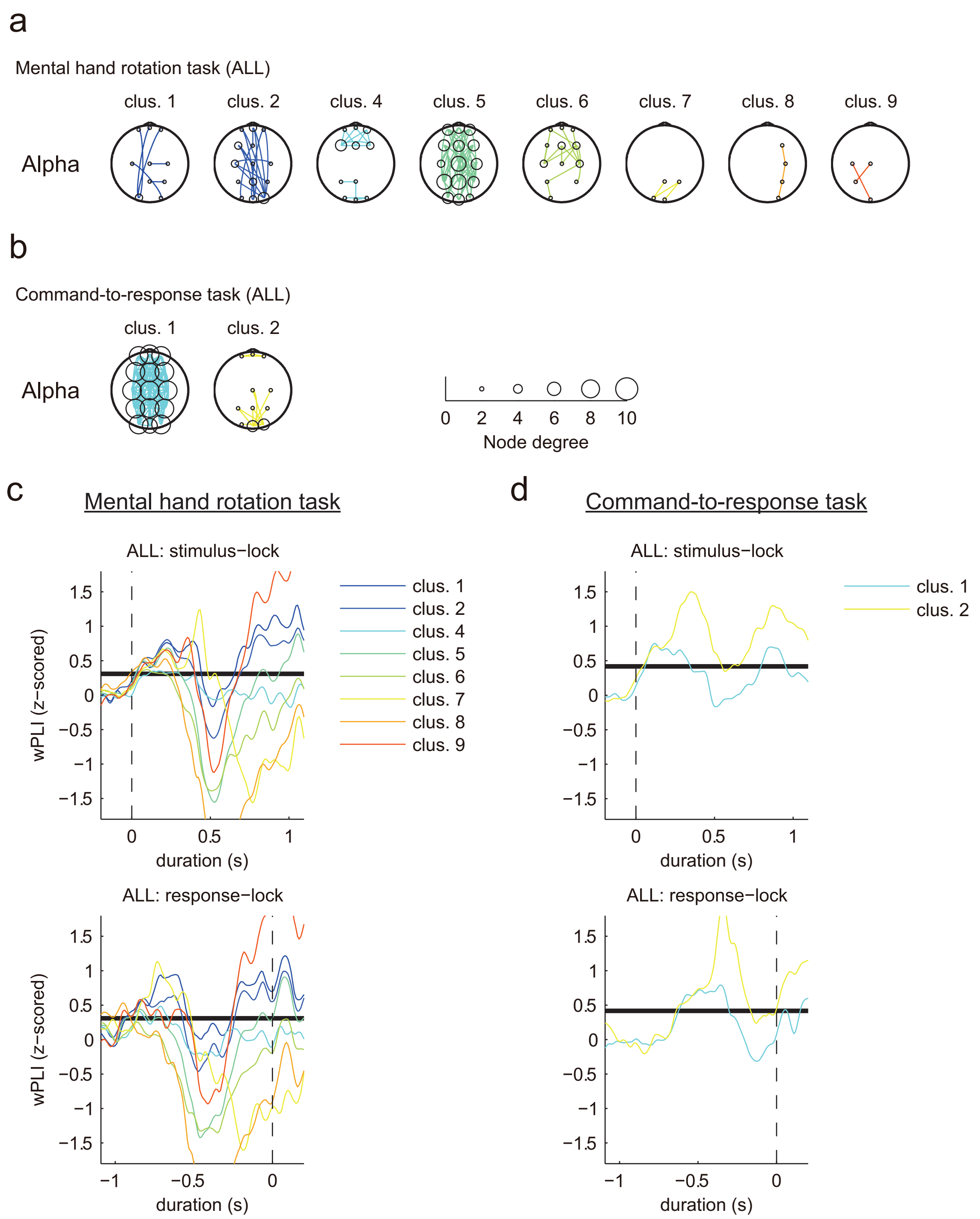

Figure S11: Estimated clusters and functional connectivity based on time series matching of z-scored wPLIs using DTW (all trial, alpha band). (a) Estimated clusters of functional connectivity for all trials in the mental hand rotation task. Eight of 10 clusters were estimated to exhibit significant connectivity. (b) Estimated significant clusters of functional connectivity for all trials in the command-to-response task. Two clusters were estimated to exhibit significant connectivity. Marker size of electrodes for each topographical location corresponded to the node degree of connectivity. (c, d) Cluster-averaged z-scored wPLIs for each task (upper panels: stimulus-locked average; lower panels: response-locked average). Bold black lines indicate a significant level of temporal change in the cluster-averaged value of phase-synchronization ($P < 0.05$ with FDR correction).

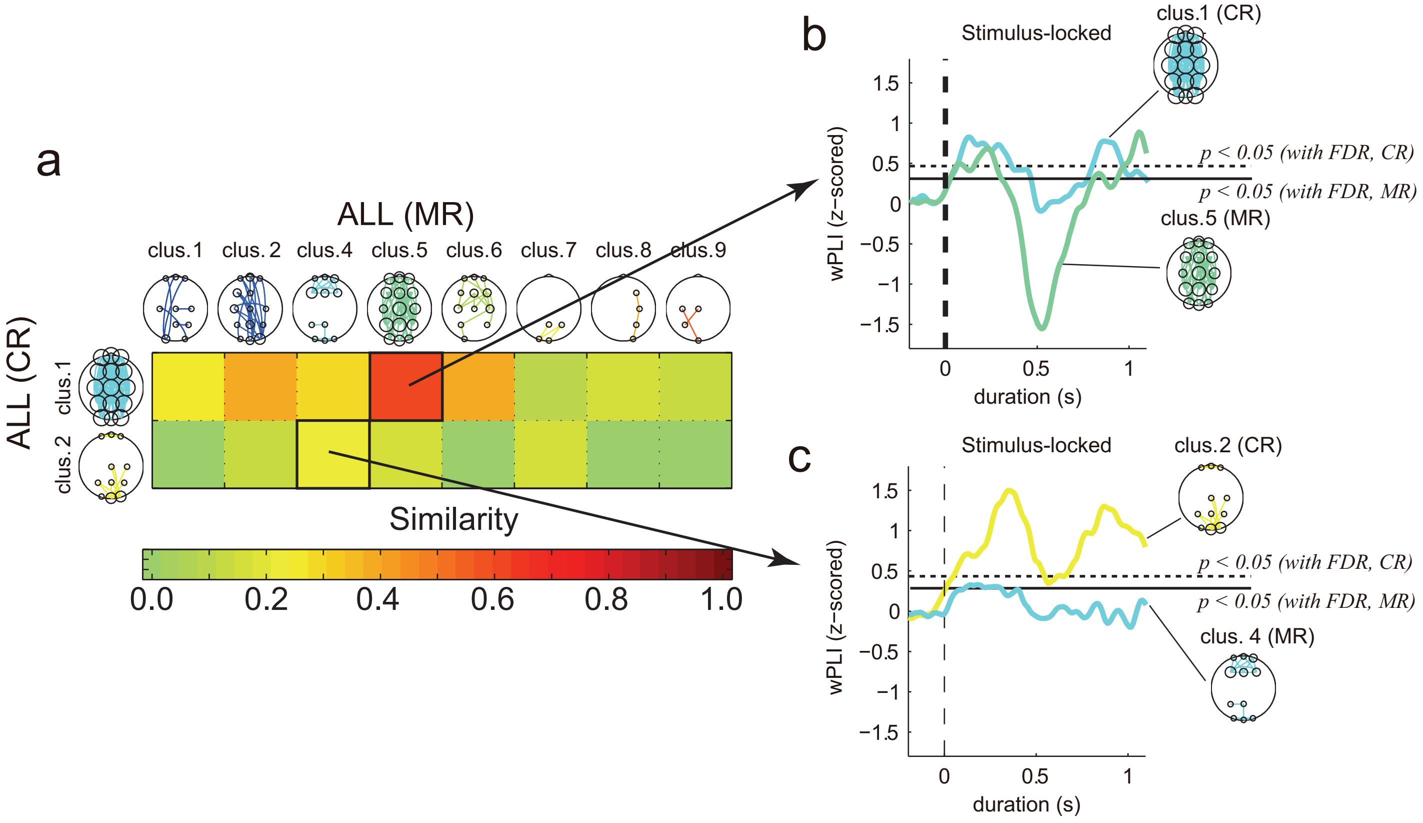

Figure S12: Comparison of cluster-by-cluster similarity of connectivity patterns (switch trials). (A) A matrix showing the cluster-by-cluster similarity between two tasks. (B, C) Pairs of clusters with high similarity and comparison of temporal changes of cluster-averaged wPLIs.

5 SUPPLEMENTARY RESULT OF PRE-PROCESSING

The average number of trials across subjects after outlier rejection is shown in Table S1. The mental hand rotation task in the current study was designed to ensure that the frequency of repeat trials was approximately 60%. In a total of 10 sessions, the visual stimuli were presented 1120 times (switch: 448 trials = 64 trials $\times$ 7 angle conditions / repeat: 672 trials = 96 trials $\times$ 7 angle conditions). Note that the frequency of stimulus presentation in each hand and angle condition was regularized to be approximately equal for each trial condition. As a result, $86.45\% \pm 5.34$ (mean $\pm$ SD) of switch trials and $89.58\% \pm 4.73$ (mean $\pm$ SD) of repeat trials were used to calculate the average for each subject in each angle condition.

Table S1. Average number of trials after artifact rejection in each condition of the presented angle. Each number indicate the average number of trial across subjects (mean $\pm$ s.e.m).

	-135°	-90°	-45°	0°	45°	90°	135°
switch	54.64±1.06	52.92±1.25	58.24±1.35	58.32±0.88	58.04±1.82	56.80±1.09	48.36±1.97
repeat	90.16±2.33	91.64±1.65	85.84±1.35	88.52±1.37	82.60±1.18	86.08±1.80	77.20±2.47

REFERENCES

Bauer, M., Kluge, C., Bach, D., Bradbury, D., Heinze, H. J., Dolan, R. J., et al. (2012). Cholinergic enhancement of visual attention and neural oscillations in the human brain. *Current Biology* 22, 397–402. doi:10.1016/j.cub.2012.01.022

Bressler, S. L., Tang, W., Sylvester, C. M., Shulman, G. L., and Corbetta, M. (2008). Top-Down Control of Human Visual Cortex by Frontal and Parietal Cortex in Anticipatory Visual Spatial Attention. *Journal of Neuroscience* 28, 10056–10061. doi:10.1523/JNEUROSCI.1776-08.2008

Caliński, T. and Harabasz, J. (1974). A dendrite method for cluster analysis. *Communications in Statistics-theory and Methods* 3, 1–27

Karamzadeh, N., Medvedev, A., Azari, A., Gandjbakhche, A., and Najafizadeh, L. (2013). Capturing dynamic patterns of task-based functional connectivity with eeg. *Neuroimage* 66, 311–317

Kuramoto, Y. (1975). Self-entrainment of a population of coupled non-linear oscillators. In *International symposium on mathematical problems in theoretical physics* (Springer), 420–422

Mars, R. B., Verhagen, L., Gladwin, T. E., Neubert, F.-X., Sallet, J., and Rushworth, M. F. (2016). Comparing brains by matching connectivity profiles. *Neuroscience & Biobehavioral Reviews* 60, 90–97

Meszlényi, R., Peska, L., Gál, V., Vidnyánszky, Z., and Buza, K. (2016). Classification of fmri data using dynamic time warping based functional connectivity analysis. In *2016 24th European Signal Processing Conference (EUSIPCO)*. 245–249. doi:10.1109/EUSIPCO.2016.7760247

Müller, M. (2007). *Information retrieval for music and motion* (Verlag Berlin Heidelberg: Springer)

Romei, V., Gross, J., and Thut, G. (2010). On the Role of Prestimulus Alpha Rhythms over Occipito-Parietal Areas in Visual Input Regulation: Correlation or Causation? *Journal of Neuroscience* 30, 8692–8697. doi:10.1523/JNEUROSCI.0160-10.2010

Sakoe, H. and Chiba, S. (1978). Dynamic programming algorithm optimization for spoken word recognition. *IEEE transactions on acoustics, speech, and signal processing* 26, 43–49

Shimaoka, D., Kitajo, K., Kaneko, K., and Yamaguchi, Y. (2010). Transient process of cortical activity during necker cube perception: from local clusters to global synchrony. *Nonlinear biomedical physics* 4, S7

Stam, C. J., Nolte, G., and Daffertshofer, A. (2007). Phase lag index: assessment of functional connectivity from multi channel eeg and meg with diminished bias from common sources. *Human brain mapping* 28, 1178–1193
